# Efficacy of Neck-Specific Exercise With Internet Support Versus Neck-Specific Exercise at a Physiotherapy Clinic in Chronic Whiplash-Associated Disorders: Multicenter Randomized Controlled Noninferiority Trial

**DOI:** 10.2196/43888

**Published:** 2023-06-20

**Authors:** Gunnel Peterson, Anneli Peolsson

**Affiliations:** 1 Centre for Clinical Research Sörmland Uppsala University Eskilstuna Sweden; 2 Department of Health, Medicine and Caring Sciences Unit of Physiotherapy Linköping University Linköping Sweden; 3 Department of Health, Medicine and Caring Sciences Occupational and Environmental Medicine Center, Unit of Clinical Medicine Linköping University Linköping Sweden

**Keywords:** internet-based intervention, telerehabilitation, whiplash associated disorders, neck, whiplash, physiotherapy, physiotherapist, physical therapy, neck pain, exercise, chronic pain, digital health intervention, telehealth, rehabilitation, pain management, internet-based, telemedicine, digital health

## Abstract

**Background:**

Neck-specific exercises (NSE) supervised by a physiotherapist twice a week for 12 weeks have shown good results in chronic whiplash-associated disorders (WADs), but the effect of exercise delivered via the internet is unknown.

**Objective:**

This study examined whether NSE with internet support (NSEIT) and 4 physiotherapy sessions for 12 weeks were noninferior to the same exercises supervised by a physiotherapist twice a week for 12 weeks (NSE).

**Methods:**

In this multicenter randomized controlled noninferiority trial with masked assessors, we recruited adults aged 18-63 years with chronic WAD grade II (ie, neck pain and clinical musculoskeletal signs) or III (ie, grade II plus neurological signs). Outcomes were measured at baseline and at 3- and 15-month follow-ups. The primary outcome was change in neck-related disability, measured with the Neck Disability Index (NDI; 0%-100%), with higher percentages indicating greater disability. Secondary outcomes were neck and arm pain intensity (Visual Analog Scale [VAS]), physical function (Whiplash Disability Questionnaire [WDQ] and Patient-Specific Functional Scale [PSFS]), health-related quality of life (EQ-5D-3L and EQ VAS), and self-rated recovery (Global Rating Scale [GRS]). The analyses were conducted on an intention-to-treat basis and with the per-protocol approach as sensitivity analyses.

**Results:**

Between April 6, 2017, and September 15, 2020, 140 participants were randomly assigned to the NSEIT group (n=70) or the NSE group (n=70); 63 (90%) and 64 (91%), respectively, were followed up at 3 months, and 56 (80%) and 58 (83%), respectively, at 15 months. NSEIT demonstrated noninferiority to NSE in the primary outcome NDI, as the 1-sided 95% CI of the mean difference in change did not cross the specified noninferiority margin (7 percentage units). There were no significant between-group differences in change in NDI at the 3- or 15-month follow-up, with a mean difference of 1.4 (95% CI –2.5 to 5.3) and 0.9 (95% CI –3.6 to 5.3), respectively. In both groups, the NDI significantly decreased over time (NSEIT: mean change –10.1, 95% CI –13.7 to –6.5, effect size=1.33; NSE: mean change –9.3, 95% CI –12.8 to –5.7, effect size=1.19 at 15 months; *P*<.001). NSEIT was noninferior to NSE for most of the secondary outcomes except for neck pain intensity and EQ VAS, but post hoc analyses showed no differences between the groups. Similar results were seen in the per-protocol population. No serious adverse events were reported.

**Conclusions:**

NSEIT was noninferior to NSE in chronic WAD and required less physiotherapist time. NSEIT could be used as a treatment for patients with chronic WAD grades II and III.

**Trial Registration:**

ClinicalTrials.gov NCT03022812; https://clinicaltrials.gov/ct2/show/NCT03022812

## Introduction

A whiplash injury is common after a motor vehicle accident, and many individuals experience chronic and disabling whiplash-associated disorders (WADs) [1-3]. Approximately 50% of individuals will not recover after a whiplash injury [1], and 30% will experience severe symptoms [4]. No evidence is currently available regarding invasive interventions [5], and exercise and patient education are recommended [6], though only modest effects have been demonstrated. In chronic WAD grades I (ie, neck pain but no physical symptoms) and II (ie, neck pain and clinical musculoskeletal signs) [7], one visit to a physiotherapist is equally effective as more comprehensive exercise programs but shows only a small treatment effect [8,9]. These results indicate that patients with chronic WAD may not need extensive visits to health care providers, but patients with worse symptoms, as in WAD grade III (ie, grade II plus neurological signs) [7], were excluded [8,9]. Neck-specific exercises (NSEs) with or without a behavioral approach have demonstrated promising results in WAD grades II and III, with long-term improvement in disability compared to prescribed physical activity [10,11]. Neck pain and impaired neck muscle function have been reported in WAD [12-14], and improved muscle function and decreased pain were seen after 3 months of NSE [15], with large effect sizes for self-reported neck function (*r*=0.88) [16]. However, NSE included 24 visits to the physiotherapist, and more efficient ways of delivering NSE are needed. Internet-based interventions can provide better access to health care, saving time and costs for both patients and society. Internet-based exercise programs have not been evaluated in neck pain and have shown inconclusive results in knee osteoarthritis [17], low back pain [18], and the enhancement of physical activity [19]. The mixed results were related to study quality, low motivation, and participants’ lack of support when relying only on internet-based care. A combination of internet and face-to-face treatment helped patients safely perform exercises with a sense of control over their symptoms [20]. A few visits to a physiotherapist can help patients with WAD safely perform the exercises and may be important for reducing pain-related fear of movement. We hypothesized that NSE delivered via the internet (IT) with 4 visits to the physiotherapist (NSEIT) was noninferior to NSE (24 physiotherapist visits). This was based on the expectation that NSEIT would be sufficient to gain the same improvements in disability as NSE is reported to have in previous studies [10,11,15,16]. The aim of this study was to evaluate whether NSE with internet support (NSEIT) and 4 physiotherapy sessions was noninferior to the same exercises supervised by a physiotherapist twice a week for 12 weeks (24 sessions).

## Methods

### Design and Setting

This prospective multicenter randomized controlled trial (RCT) compared 2 groups: NSEIT for 12 weeks with 4 visits to the physiotherapist (NSEIT group) versus supervised NSE twice a week for 12 weeks (NSE group).

### Ethics Approval

The study was approved by the regional ethical review board in Linköping, Sweden (2016/135-31) and followed the Declaration of Helsinki. The study protocol has been published elsewhere [21], and the trial is registered with ClinicalTrials.gov (NCT03022812).

### Participants and Randomization

Study information was provided to potential participants through reports in newspapers and the media, and individuals interested in participating in the study completed a small web-based survey. An apparently eligible participant was contacted for a telephone interview to further verify their medical history and ability to participate in the exercise program. The last step in recruiting participants was a physical examination conducted by a test leader (a physiotherapist) to ensure that the criteria for study participation were met. Informed written consent was obtained from all trial participants, and they filled out a baseline questionnaire. The participants in this noninferiority trial were very similar to the participants in the studies that established efficacy in NSE [10,11,15,16]. The only difference was that “months since injury” were lower in the previous studies (mean 19, SD 8.7 [10,11], mean 20, SD 8.3 [15], median 18, IQR 14-26 [16]), compared with this study’s mean of 27.4 (SD 21.0). The reason for the difference was that participants could be included up to 5 years after the accident in this study, compared with 3 years in the previous studies. Individuals fulfilling the following criteria were eligible for inclusion: persistent neck pain and disability after a whiplash injury in a 4-wheeled motor vehicle traffic accident at least 6 months, but less than 5 years, ago; WAD corresponding to grade II (neck pain, stiffness, tenderness, and clinical musculoskeletal signs) or III (grade II plus neurological signs); age between 18 and 63 years; average estimated neck pain in the last week at least 20 mm on the Visual Analog Scale (VAS); neck disability >20% on Neck Disability Index (NDI); daily access to a computer, tablet, or smartphone and the internet; time to follow the treatment program; and neck pain, neck stiffness, or cervical radiculopathy within the first week after the injury. Exclusion criteria were signs of head injury at the time of the whiplash injury, including loss of consciousness, amnesia before or after the injury, altered mental status (eg, confusion or disorientation), or focal neurological changes in smell and taste. Additional exclusion criteria were previous fractures or dislocation of the cervical spine; known or suspected serious physical pathology, including myelopathy; spinal tumors, spinal infection, or ongoing malignancy; previous severe neck problems that resulted in sick leave for more than a month in the year prior to the current whiplash injury; cervical spine surgery; generalized or more dominant pain elsewhere in the body; other illness or injury that may prevent full participation; inability to understand and write in Swedish; diagnosed severe mental illness, such as psychosis, schizophrenia, or personality disorders; current alcohol and drug abuse; or participation in the earlier NSE study [10].

A computer-based block randomization list stratified by sex was used for randomization to the 2 groups and allocated by a project team member (GP) not otherwise involved in any of the tests or treatments. After the test leader confirmed eligibility and baseline data were collected, GP sent the participant’s group allocation and contact details in a sealed, opaque envelope to the treating physiotherapist, who called the participant to book an appointment. The treating physiotherapist worked in a health care center near the participant’s home or workplace. This was aimed at facilitating the participant’s opportunity to take part in the study. An envelope was sent to the participants with information on their group allocation and a reminder to contact GP in 2 weeks if the physiotherapist had not called. The test leader was blinded to group allocation at baseline and at the 3- and 15-month follow-ups. The participant was blinded to group allocation when baseline data were collected. At 3 and 15 months, the participants reported adverse events to the test leader (defined as an unforeseen or dangerous reaction to the exercise intervention) or side effects (defined as commonly increased symptoms related to exercises, such as muscle soreness or temporally increased pain, dizziness, or headache for <2 weeks). An independent statistician analyzed the coded data.

### Interventions

Both interventions are described in Multimedia Appendices 1 and 2.

The NSEIT group had 4 sessions at the physiotherapy clinic. The first session included a clinical examination and an introduction to the first exercises. In the follow-up sessions (weeks 2, 3, and 7), new exercises were introduced, progressed, and followed up to ensure correct performance. Participants had access to the internet-based program on a website. The program included information, photos, and videos of all exercises, with clear stepwise progression and an exercise diary. The NSEs were initially targeted to activate the deep neck muscle layers with an individual progression of endurance exercises within the participant’s symptom tolerance. The participants could contact the physiotherapist by telephone or book an appointment if pain or other symptoms increased during the home exercise period.

The NSE group received the same information and exercise program as the NSEIT group, but it was delivered by a physiotherapist. The participants attended 2 sessions a week for 12 weeks at a physiotherapy clinic. In both groups, exercise-related neurological pain was not acceptable and temporarily increased muscle soreness after exercises was only allowed if it did not increase neck pain over time (Multimedia Appendices 1 and 2).

After the 12 weeks, participants in both groups were encouraged to continue training on their own 2-3 times a week in accordance with the 2017 World Health Organization (WHO) guidelines [22] and to include NSEs in their training program.

Ventral neck muscle endurance is considered important for recovery from neck pain and disability, but relatively small improvements were seen in the previous RCT [11]. Therefore, 2 ventral neck exercises were added in this study after 6-7 weeks of exercises. Except for that, the neck-specific program in the NSE and NSEIT groups was similar to the exercises and information in the RCTs [10,11,15,16]. Before the study started, the physiotherapists received 1 day of theoretical and practical training from the project leaders. The physiotherapists could contact the project leaders if they required further advice regarding the interventions.

### Outcomes

The outcome measures were reported using questionnaires on Linköping University’s website, Survey and Reports, at baseline and 3 and 15 months after baseline (ie, 12 months after the intervention ended). Ratings on a VAS and Patient-Specific Functional Scale (PSFS) were collected by the test leader. The participants formulated 3 specific activity goals to improve daily activities at work, during leisure time, and during physical exercise using the PSFS. The goals were specified for time and designed to be achievable during the 3-month rehabilitation program (eg, computer work for 20 minutes, 5 days a week).

The null hypothesis was that the clinically important reduction in the NDI in the NSE group would be at least 7 points (percent scale) better than the reduction in the NSEIT group. The noninferiority margin was based on the recommendation for a minimal clinically important change in NDI (7%) [23,24]. These values were also based on the SD (13.4) of the NDI in a previous study of NSEs in individuals with chronic WAD of grades II and III [10].

The primary outcome was the NDI, which is considered a reliable and valid measurement of disability due to neck pain [23]. The NDI includes 10 items of neck-related disability, graded from 0 (no activity limitations) to 5 (major activity limitations), which are summed and transformed into a percentage (0%, no pain or disability; 100%, highest score for pain and disability). Secondary outcomes were average neck and arm pain during the previous week and neck and arm pain now, which were measured using a VAS from 0 (no pain) to 100 (worst imaginable pain) [25]. Disability was also measured with the Whiplash Disability Questionnaire (WDQ) using a score of 0 (no disability) to 130 (major disability) [26]. Change in physical function was assessed using a PSFS graded from 0 (unable to do) to 10 (functional level equal to preinjury status) [27]. Quality of life was measured with the EQ-5D-3L index and EQ VAS [28], and self-rated recovery was measured with the 11-point Global Rating Scale (GRS; –5=vastly worse, 0=unchanged, +5=completely recovered) [29]. The outcomes in this noninferiority trial are very similar to those in the previous RCT [10,11], establishing the efficacy of the NSE program. In this study, 2 patient-reported outcomes, WDQ and GRS, were added to collect patient-centered data that may provide improved information on the impact of a medical condition and its treatment from the patient's perspective.

### Statistical Analysis

Sample size and power calculations were carried out using PASS (Power Analysis and Sample Size) software (version 13.0.8; NCSS, LLC) based on the primary outcome NDI [23,24]. To detect a between-group noninferiority margin of 7 (percentage units) with 1-sided *α*=.025 and *β*=.8, a total of 47 participants were needed in each group. To account for attrition, 70 participants were included in each group. Based on previous research, the noninferiority margins for the secondary outcomes were a 10-mm reduction in pain on the VAS [25], a 15-point reduction on the WDQ [26], a 2-point change in function on the PFSF [27], a 0.1 unit change in quality of life on the EQ-5D-3L index, a 10 units change on the EQ VAS [28], and a 2-point change in self-rated recovery on the GRS [29]. For comparison purposes, the between-group effects (mean difference in change between baseline to 3 months and baseline to 15 months) and the noninferiority margins of primary and secondary outcomes were standardized. The standardized between-group effect, that is, effect size at 3 and 15 months, was calculated as the mean differences between NSEIT and NSE divided by the pooled standard deviation (the weighted average of each group’s standard deviation). The standardized margin was calculated as the predefined noninferiority margin divided by the pooled standard deviation.

The noninferiority tests on primary and secondary outcomes were performed in R (version 3.6.0; R Foundation for Statistical Computing), using the package *equivUMP* (version 0.1.1). NSEIT is considered noninferior to NSE if the lower limit (the 1-sided 95% CI of the effect size) does not exceed the standardized margin. Post hoc analyses were conducted with linear mixed models (3 time points: baseline, 3 months, and 15 months × 2 groups: NSEIT and NSE), using a restricted maximum likelihood estimate, allowing all participants with at least one observation to be included in the analysis. Unstructured covariance was used for the repeated measures. The analyses were conducted on an intention-to-treat (ITT) basis and repeated, for sensitivity reasons, for the per-protocol population. The per-protocol population comprised participants who were compliant with exercise (defined as at least 50% self-reported attendance to exercises).

Demographic characteristics are presented as mean and SD or median and IQR. For binary baseline data, *χ*^2^ tests were used.

The proportion of responders to treatment was determined in each group, with clinically important improvements in NDI (≥7% reduction) [23,24], VAS (≥50% reduction in individuals with baseline levels ≥10 mm) [25], and PSFS (≥2-point improvement) [24]. NDI classification, following the classification used by Vernon and Mior [30]. The inclusion criterion was NDI >20%; consequently, the mild disability class at baseline contained participants over that limit. Effect sizes were categorized with Cohen *d*=0.5 representing a medium effect size and *d*=0.8 representing a large effect size.

### Patients and the Public

Patients were involved in developing the questionnaires. Before recruitment to the study started, the questionnaires were tested by patients to ensure readability and the inclusion of relevant questions. Patient satisfaction with the exercises, information, tests, and their total experience with the interventions were measured post intervention. Qualitative interviews were conducted with a proportion of patients after the study [31], and patients have been involved in the further development of the internet-based program.

## Results

### Principal Findings

**Figure 1:**
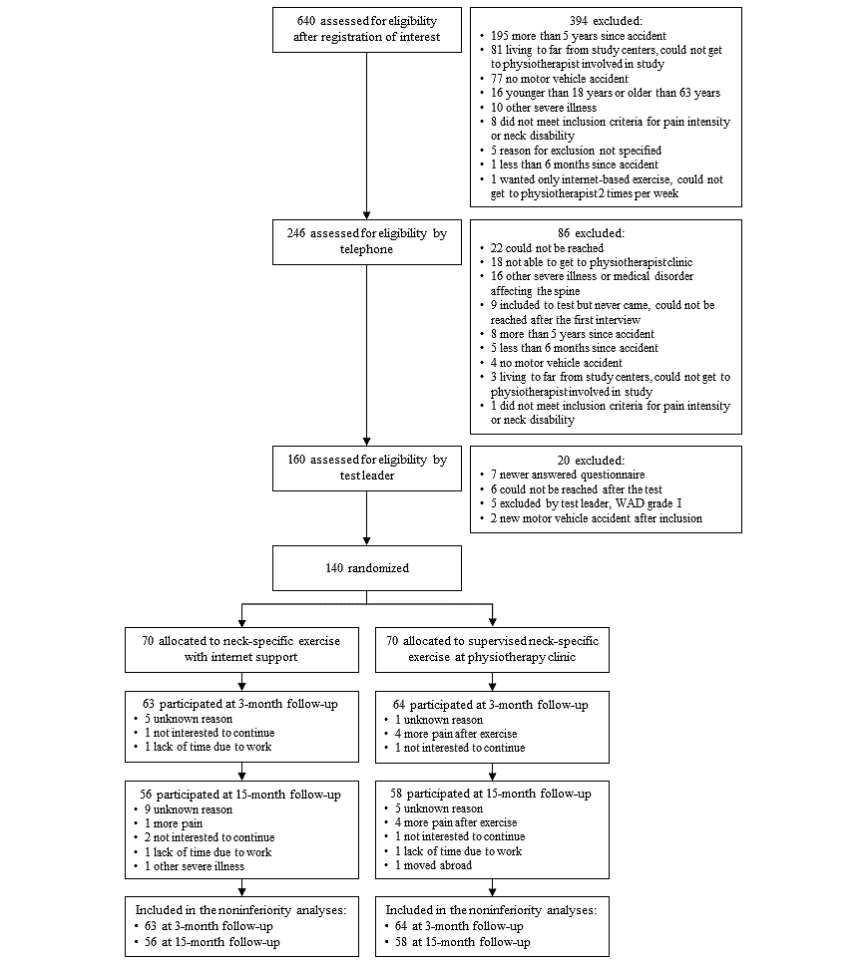
Flowchart of participants in the study. WAD: whiplash-associated disorder.

The participants were recruited from 10 county councils in Sweden between April 6, 2017, and September 15, 2020, with a 3- and 15-month follow-up. A total of 140 participants were included and randomized to either the NSEIT (n=70) or NSE (n=70) groups. In the NSEIT group, 63 (90%) and 56 (80%) participants were followed up at 3 and 15 months, respectively. In the NSE group, 64 (91%) and 58 (83%) participants were followed up at 3 and 15 months, respectively (Figure 1). Of the 140 participants, 29 (21%) had mild disability (NDI: >20%-28%), 88 (63%) had moderate disability (NDI: 30%-48%), and 23 (16%) had severe disability (NDI: 50%-68%) at baseline. At the 15-month follow-up, 14 (12%) individuals reported no disability (NDI: 0%-8%), 42 (37%) mild disability (NDI: 10%-28%), 46 (41%) moderate disability (NDI: 30%-48%), and 11 (10%) severe disability (NDI: 50%-68%), with no significant difference between the exercise groups (*P*=.20).

The participants’ self-reported compliance with exercise was higher in the NSE group (59/63, 94%) than in the NSEIT group (46/61, 75%, *P*=.006) during the 12-week intervention period. The trial interventions were delivered by 57 physiotherapists from 10 county councils. The NSE group attended a median of 20 (IQR 14-23) of the 24 maximum sessions at the physiotherapy clinic. In the NSEIT group, the median number of sessions was 4 of a maximum of 4 (IQR 4-4) sessions. There were no significant differences in baseline variables (Table 1) between the groups (*P*≥.15).

**Table 1:** Baseline descriptive characteristics of trial participants, by treatment group.

Baseline characteristic			NSEIT^a^ (n=70)	NSE^b^ (n=70)
Age (years), mean (SD)			40.4 (11.6)	40.5 (11.4)
Months since injury, mean (SD)			27.4 (21.0)	25.2 (15.5)
**WAD^c^, n (%)**				
	Grade II		46 (66)	43 (61)
	Grade III		24 (34)	27 (39)
Sex (female), n (%)			55 (79)	55 (79)
**Educational level, n (%)**				
	Elementary		1 (1)	0 (0)
	High school		30 (43)	39 (57)
	University		35 (50)	27 (39)
	Other		4 (6)	3 (4)
Marital status, married or cohabiting (yes), n (%)			52 (74)	53 (76)
Previous treatment (yes), n (%)			62 (89)	66 (94)
**Previous physiotherapy treatments, n (%)**				
	Advice		30 (43)	23 (33)
	Neck exercises^d^		37 (53)	36 (51)
	General exercises		15 (21)	12 (17)
	Acupuncture		10 (14)	9 (13)
	Other (eg, massage, yoga, and manipulation)		5 (7)	12 (17)
**Expectation to participate in the study, n (%)**				
	Fully recovered		3 (4)	5 (7)
	Much improved		51 (73)	49 (70)
	Some relief		14 (20)	15 (22)
	No expectations		2 (3)	1 (1)
Use of analgesic drugs (yes), n (%)			57 (81)	59 (84)
**Compensation, n (%)**				
	No		22 (31)	15 (21)
	Yes		29 (42)	42 (60)
	Not decided		19 (27)	13 (19)
**Present employment status, n (%)**				
	**Employed**			
		Full-time	38 (54)	44 (63)
		Part-time	14 (20)	12 (17)
	**Self-employed**			
		Full-time	3 (4)	4 (6)
		Part-time	6 (8)	2 (3)
	Student		7 (10)	6 (8)
	Unemployment compensation		2 (3)	2 (3)
	**Sick leave**			
		Full-time	3 (4)	3 (4)
		Part-time	4 (6)	4 (6)

^a^NSEIT: neck-specific exercise with internet support.

^b^NSE: neck-specific exercise at a physiotherapy clinic.

^c^WAD: whiplash-associated disorder.

^d^Other neck exercises than the neck-specific exercises in this study.

### Between-Group Differences

Baseline, 3-month, and 15-month outcomes are listed in Table 2, and between-group comparisons are in Table 3. The NSEIT group demonstrated noninferiority to the NSE group in the primary outcome (Table 3). The post hoc analyses demonstrated no mean group differences in the NDI of 1.4 (95% CI –2.5 to 5.3) at 3 months and 0.9 (95% CI –3.6 to 5.3) at 15 months (Table 4). Furthermore, NSEIT was noninferior to NSE for all secondary outcomes except neck pain intensity at 3 and 15 months and EQ VAS at 15 months, in favor of the NSE group (Table 3). The post hoc analyses demonstrated no between-group differences (*P*≥.20): mean differences were –4.4 (95% CI –12.7 to 4.0) for neck pain now at 3 months, –6.1 (95% CI –14.4 to 2.2) at 15 months, and 5.4 (95% CI –2.4 to 13.2) for EQ VAS at 15 months (Table 4). Similar results were seen in the per-protocol population. NSEIT was noninferior to NSE in primary and secondary outcomes, except for neck pain intensity and EQ-VAS, but post hoc analyses demonstrated no group differences (Multimedia Appendix 3).

**Table 2:** Primary and secondary outcomes for each treatment group at baseline and at the 3- and 15-month follow-up.

		Baseline, mean (SD)			3 months, mean (SD)			15 months, mean (SD)	
		NSEIT^a^ (n=70)	NSE^b^ (n=70)	NSEIT (n=63)		NSE (n=64)	NSEIT (n=56)		NSE (n=58)
**Primary outcome**									
	NDI^c^ (%)^d^	39.4 (12.2)	36.6 (10.8)	30.5 (15.2)		29.0 (13.8)	28.6 (16.3)		27.6 (13.4)
**Secondary outcome**									
	Neck pain average^e^	44.9 (20.6)	47.6 (21.7)	30.7 (24.6)		34.2 (24.9)	27.1 (24.5)		28.1 (22.7)
	Neck pain now^e^	34.6 (21.4)	39.7 (22.4)	24.6 (22.9)		25.7 (23.6)	22.5 (22.1)		22.4 (20.2)
	Arm pain average^f^	22.0 (23.3)	19.9 (23.5)	12.7 (16.6)		16.3 (21.8)	13.2 (21.8)		11.6 (20.4)
	Arm pain now^f^	16.7 (21.0)	15.5 (21.5)	10.5 (16.9)		15.8 (23.6)	10.7 (18.5)		10.0 (18.4)
	WDQ^g,h^	56.7 (21.7)	56.8 (22.4)	47.2 (27.2)		43.8 (24.4)	39.9 (27.2)		38.3 (24.7)
	PSFS^i^ work^j^	3.9 (2.5)	3.9 (2.2)	5.8 (2.7)		5.6 (2.4)	6.8 (2.4)		6.3 (2.4)
	PSFS leisure time^j^	3.7 (2.1)	3.6 (2.3)	5.9 (2.7)		5.9 (2.4)	7.1 (2.7)		6.6 (2.9)
	PSFS physical activity^j^	3.2 (2.5)	3.5 (2.8)	5.1 (3.1)		4.7 (3.4)	7.1 (2.9)		6.2 (3.2)
	EQ-5D-3L index^k^	0.57 (0.29)	0.64 (0.21)	0.63 0.27)		0.67 (0.23)	0.66 (0.25)		0.72 (0.23)
	EQ VAS^l,m^	57.7 (18.7)	58.6 (17.0)	63.5 (20.9)		65.9 (20.3)	62.9 (22.8)		69.2 (19.6)
	GRS^n,o^	N/A^p^	N/A	1.8 (1.7)		1.7 (1.8)	2.1 (1.6)		1.9 (1.9)

^a^NSEIT: neck-specific exercise with internet support.

^b^NSE: neck-specific exercise at a physiotherapy clinic.

^c^NDI: Neck Disability Index.

^d^Scored from 0% (no disability) to 100% (high disability).

^e^Neck pain average previous week and now; Visual Analog Scale, scored from 0 mm (no pain) to 100 mm (worst imaginable pain).

^f^Arm pain average previous week and now; Visual Analog Scale, scored from 0 mm (no pain) to 100 mm (worst imaginable pain).

^g^WDQ: Whiplash Disability Questionnaire.

^h^Scored from 0 (no disability) to 130 (high disability).

^i^PSFS: Patient-Specific Functional Scale.

^j^Scored from 0 (unable to do) to 10 (functional level equal to preinjury status).

^k^Scored from –0.59 to 1, where 1 indicating “full health” and 0 “a state as bad as being dead.”

^l^VAS: Visual Analog Scale.

^m^Scored from 0 (the worst imaginable health state) to 100 (the best imaginable health state).

^n^GRS: Global Rating of Change Scale.

^o^Scored from –5 (vastly worse), 0 (unchanged), to 5 (completely recovered).

^p^N/A: not applicable.

**Table 3:** Noninferiority analyses on standardized between-group effects in primary and secondary outcomes at 3 and 15 months. Positive values indicate favorable change in neck-specific exercise with internet support (NSEIT) compared to neck-specific exercise at a physiotherapy clinic (NSE).

		Standardized between-group effects at 3 months				Standardized between-group effects at 15 months		
		Effect size	95% CI lower limit	Standardized margin^a^	Effect size		95% CI lower limit	Standardized margin^a^
**Primary outcome**								
	NDI^b^ (%)^c^	0.129	–0.164	–0.628	0.099		–0.211	–0.581
**Secondary outcome**								
	Neck pain average^d^	–0.016	–0.317	–0.409	–0.144		–0.473	–0.443
	Neck pain now^d^	–0.173	–0.474	–0.423	–0.187		–0.516	–0.451
	Arm pain average^e^	0.218	–0.088	–0.453	–0.018		–0.351	–0.426
	Arm pain now^e^	0.282	–0.025	–0.505	–0.020		–0.352	–0.468
	WDQ^f,g^	–0.133	–0.425	–0.706	–0.113		–0.423	–0.739
	PSFS^h^ work^i^	0.158	–0.147	–0.748	0.122		–0.218	–0.672
	PSFS leisure time^i^	0.100	–0.204	–0.723	0.071		–0.263	–0.604
	PSFS physical activity^i^	0.167	–0.143	–0.586	0.162		–0.188	–0.586
	EQ-5D-3L index^j^	0.189	–0.104	–0.449	–0.010		–0.322	–0.409
	EQ VAS^k,l^	–0.034	–0.329	–0.434	–0.243		–0.556	–0.470

^a^Standardized margin indicates the standardized noninferiority margin. NSEIT is considered noninferior to NSE if the lower limit (the 1-sided 95% CI of the effect size) does not exceed the standardized margin.

^b^NDI: Neck Disability Index.

^c^Scored from 0% (no disability) to 100% (high disability). Noninferiority margin of 7% units.

^d^Neck pain average previous week and now; Visual Analog Scale, scored from 0 mm (no pain) to 100 mm (worst imaginable pain). Noninferiority margin of 10 mm.

^e^Arm pain average previous week and now; Visual Analog Scale, scored from 0 mm (no pain) to 100 mm (worst imaginable pain). Noninferiority margin of 10 mm.

^f^WDQ: Whiplash Disability Questionnaire.

^g^Scored from 0 (no disability) to 130 (high disability). Noninferiority margin of 15 points.

^h^PSFS: Patient-Specific Functional Scale.

^i^Scored from 0 (unable to do) to 10 (functional level equal to preinjury status). Noninferiority margin of 2 points.

^j^Scored from –0.59 to 1, where 1 indicating “full health” and 0 “a state as bad as being dead.” Noninferiority margin of 0.1 units.

^k^VAS: Visual Analog Scale.

^l^Scored from 0 (the worst imaginable health state) to 100 (the best imaginable health state). Noninferiority margin of 10 units.

**Table 4:** Between-group effects (95% CI) in primary and secondary outcomes at 3- and 15 months. Positive values indicate favorable change in NSEIT compared to NSE.

			Change at 3 months				Change at 15 months					Main effect of time × group		
			NSEIT^a^-NSE^b^	*P* value		NSEIT-NSE			*P* value		*F* value^c^			*P* value
**Primary outcome**														
	NDI^d^ (%)^e^	1.4 (–2.5 to 5.3)		.47	0.9 (–3.6 to 5.3)			.70		0.26			.77	
**Secondary outcome**														
	Neck pain average^f^	0.2 (–8.5 to 8.9)		.30	–3.3 (–11.9 to 5.3)			.15		0.24			.63	
	Neck pain now^f^	–4.4 (–12.7 to 4.0)		.96	–6.1 (–14.4 to 2.2)			.45		0.29			.59	
	Arm pain average^g^	6.1 (–1.6 to 13.9)		.06	0.4 (–8.4 to 9.3)			.93		0.07			.79	
	Arm pain now^g^	6.6 (–0.4 to 13.6)		.11	–0.3 (–8.2 to 7.5)			.92		0.08			.77	
	WDQ^h,i^	–3.0 (–10.4 to 4.4)		.43	–3.1 (–10.6 to 4.3)			.41		0.28			.60	
	PSFS^j^ work^k^	0.3 (–0.7 to 1.3)		.54	0.5 (–0.6 to 1.6)			.35		0.69			.41	
	PSFS leisure time^k^	0.2 (–0.8 to 1.2)		.71	0.5 (–0.8 to 1.7)			.44		0.51			.47	
	PSFS physical activity^k^	0.8 (–0.5 to 2.0)		.23	1.1 (–0.2 to 2.4)			.10		0.59			.44	
	EQ-5D-3L index^l^	0.04 (–0.04 to 0.12)		.29	0.01 (–0.08 to 0.10)			.88		2.36			.13	
	EQ VAS^m,^^n^	–1.3 (–9.4 to 6.8)		.75	–5.4 (–13.2 to 2.4)			.17		1.39			.24	

^a^NSEIT: neck-specific exercise with internet support.

^b^NSE: neck-specific exercise at a physiotherapy clinic.

^c^Repeated measures analysis baseline to 3 and 15 months.

^d^NDI: Neck Disability Index.

^e^Scored from 0% (no disability) to 100% (high disability).

^f^Neck pain average previous week and now; Visual Analog Scale, scored from 0 mm (no pain) to 100 mm (worst imaginable pain).

^g^Arm pain average previous week and now; Visual Analog Scale, scored from 0 mm (no pain) to 100 mm (worst imaginable pain).

^h^WDQ: Whiplash Disability Questionnaire.

^i^Scored from 0 (no disability) to 130 (high disability).

^j^PSFS: Patient-Specific Functional Scale.

^k^Scored from 0 (unable to do) to 10 (functional level equal to preinjury status).

^l^Scored from –0.59 to 1, where 1 indicating “full health” and 0 “a state as bad as being dead.”

^m^VAS: Visual Analog Scale.

^n^Scored from 0 (the worst imaginable health state) to 100 (the best imaginable health state).

### Within-Group Differences

Within-group differences and the proportion of responders in self-rated recovery are shown in Multimedia Appendix 3. Both groups had a significant reduction in the NDI over time: NSEIT, mean –9.1, 95% CI –12.3 to –6.0 and mean –10.1, 95% CI –13.7 to –6.5; effect size=1.33, at 3 and 15 months, respectively; and NSE, mean –7.7, 95% CI –10.9 to –4.6 and mean –9.3, 95% CI –12.8 to –5.7; effect size=1.19, at 3 and 15 months, respectively (*P*<.001). Most of the secondary outcomes were significantly improved in both groups, with a medium to large effect (effect size=0.43-1.60, *P*=.045 to *P*<.001) (Multimedia Appendix 3).

**Figure 2:**
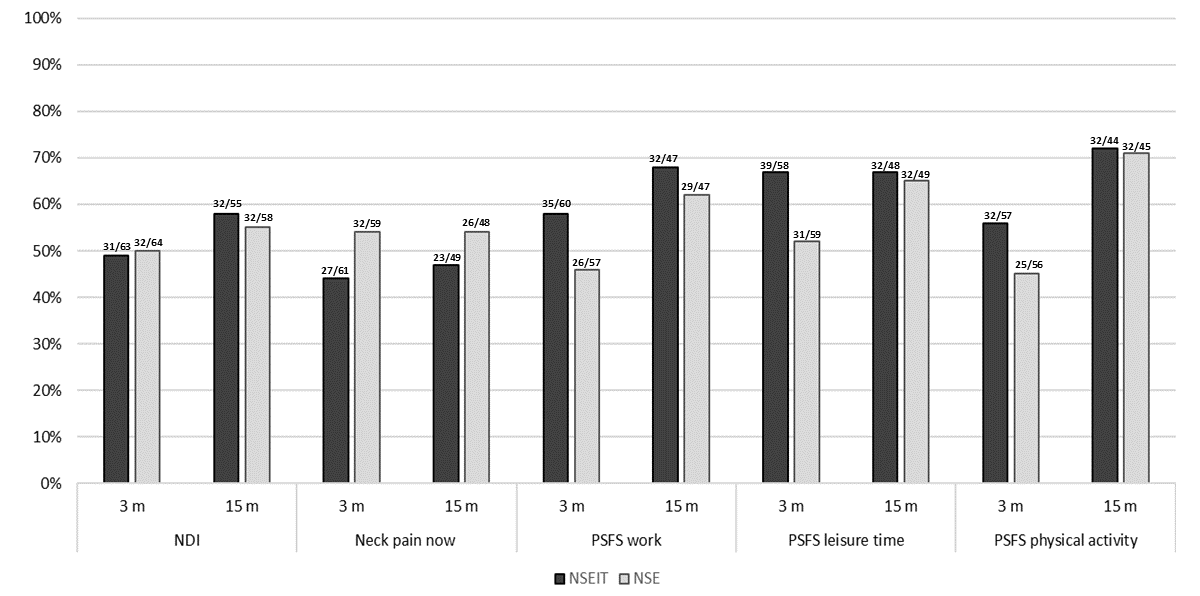
Proportion of responders to treatment at 3- and 15-month follow-up. Data labels above the bar show the absolute number and the total number of participants (absolute/total) for each measurement. The proportion of responders achieving a clinically important difference in the NDI (≥7%), Visual Analog Scale (≥50% neck pain reduction), and PSFS (≥2 points improvement in PSFS work, leisure time, and physical activity). NDI: Neck Disability Index; NSE: neck-specific exercise at a physiotherapy clinic; NSEIT: neck-specific exercise with internet support; PSFS: Patient Specific Functional Scale.

There were no between-group differences in the proportion of responders to treatment (*P*=.21 to *P*=.10). Between 31 out of 63 participants (49%) and 32 out of 55 (58%) met the criteria for a clinically important change in NDI. Between 27 out of 61 (44%) and 26 out of 48 (54%) of the participants reported a ≥50% reduction in neck pain at the 3- and 15-month follow-ups (Figure 2). The proportion of responders for the PSFS varied between 45% (25/56) and 67% (39/58) at 3 months of follow-up and was sustained or improved at the 15-month follow-up (Figure 2) and up to 73% (32/44) was sustained or improved at the 15-month follow-up (Figure 2).

### Adverse Events

No adverse events were reported. Side effects were reported by 31 participants (44%) in the NSEIT group and 39 participants (56%) in the NSE group (*P*=.60). Side effects were mainly a temporary increase in symptoms (<2 weeks) related to the progression in exercise, but 3 participants in the NSEIT group and 2 in the NSE group reported enhanced pain for >2 weeks (Multimedia Appendix 3). The symptoms decreased with a slower progression and an extended time period for performing isometric exercises in the supine position.

## Discussion

### Principal Findings

NSEIT was noninferior to NSE, and both groups demonstrated decreased disability and pain, with 47% (23/49)-58% (32/55) of the participants having a sustained clinically important change in disability and a ≥50% reduction in neck pain at the 15-month follow-up. The trial was the first to compare the efficacy of an internet-delivered NSE program including 4 physiotherapy sessions (NSEIT) with that of traditional face-to-face exercise sessions (NSE), showing sustained improved function in chronic WAD of grades II to III in both groups. The NSEIT program responds to the need for increased flexibility and availability for patients with chronic WAD.

### Comparison to Other Studies

The present findings confirm results from other studies showing that an internet-based program may be as good as face-to-face visits in health care [20,32,33]. An internet-based intervention combined with a few visits to the physiotherapist was preferable in vestibular rehabilitation [20], and the visits were important for the patient to feel safe, reassured, and motivated.

To our knowledge, no studies have investigated if assessment, diagnosis, and the progression of exercises over the internet with or without face-to-face visits can be preferable in chronic WAD grades II and III or in nonspecific neck pain. Four physiotherapy sessions were included in NSEIT during the 12-week intervention. In the first session, the physiotherapist conducted a clinical examination. Based on their examination, they customized an exercise program from a well-defined set of NSEs. The purpose was to justify the exercise program and to help the patient feel safe and motivated to perform the exercises. The other 3 sessions were aimed at answering potential questions regarding the digital information, introducing new exercises, making progress with the existing exercises, or adjusting them to a lower intensity level if needed.

To our knowledge, no previous study has investigated exercise interventions or clinical examinations over the internet for individuals with neck pain, but they have been evaluated in other musculoskeletal disorders.

Exercise interventions or clinical examinations over the internet have been evaluated for other musculoskeletal disorders. Although patients were satisfied with the assessment and diagnosis of shoulder and lower-limb disorders over the internet [34,35], they preferred face-to-face assessment. Relatively poor agreement results were also seen for the assessment of the nervous system [34]. Face-to-face sessions for patients with WAD may be important for assessment, the progression of exercises, and follow-up, especially for patients with neurological symptoms as in WAD grade III. The recovery from chronic whiplash is complex, but a positive patient and physiotherapist relationship has been identified as important in facilitating recovery [36]. The results of this study showed that NSEIT was noninferior to NSE, indicating that visits to a physiotherapist could be reduced in chronic WAD. Further research should focus on the optimal number of face-to-face visits and evaluate if digital assessment in WAD grades II and III can be conducted safely.

The effects of NSEIT on neck disability, function, and pain were at least as beneficial as NSE immediately post intervention and at 15-month follow-up. No adverse events were assigned to NSEIT, indicating that an internet-delivered exercise program with a few physiotherapy sessions was safe in WAD grades II and III. This noninferiority study confirms and extends knowledge from our previous results [10,16] that demonstrated improvements in WAD grades II and III. This strengthens the novelty and importance of this study.

Evidence of optimal treatment for chronic WAD is scarce. Although exercise has the most convincing evidence in regard to neck pain, studies of exercise interventions in chronic WAD have shown disparate results [8-10]. The discrepancy may be related to differences in inclusion criteria or exercise interventions. Previous trials included participants with no physical signs at clinical examination (WAD grade 1) [8,9], and, for those patients, advice may be sufficient. Moreover, individuals who still had neurological signs (WAD grade III) at the start of the study (3 to 12 months after the whiplash injury) were excluded [9]. Our prior studies [10,11,15,16] showed that NSE successfully improved pain and function in WAD grades II and III, whereas other exercise programs have shown meager results [8,9,37]. However, comparisons between this study and other trials [8,9,37] are difficult due to differences in, for example, inclusion criteria, patient characteristics, and methodology. The deep neck muscle layers that surround the cervical spine contribute to controlling the intersegmental joint motion [38], and dysfunction of neuromuscular control has been reported in individuals with WAD [13,39,40]. The first weeks of exercises in the NSE and NSEIT programs were aimed to facilitate and improve endurance in the deep neck-muscle layers, which may have been beneficial for these patients with chronic WAD grades II and III. However, despite the same effect of the exercise interventions in both groups, the compliance with exercise was lower in the NSEIT group, suggesting that patients with WAD can exercise at a lower level than commonly recommended (daily for the first few weeks, then proceed to 3 times a week). Further studies are required to investigate the optimal dosage.

### Strengths and Limitations of the Study

The trial was a multicenter study involving 57 physiotherapists working in primary care clinics in 10 county councils, which enhanced the risk of less control over the delivered interventions. However, the physiotherapists received theoretical and practical training, including written information, and they could contact the project leaders for support when needed. The advantage of the multicenter study is related to the fact that the interventions were delivered by physiotherapists without expert knowledge and skills in WAD and NSE. This may indicate that the results are generalizable and easy to implement in clinical practice. The compliance with exercise was lower in the NSEIT group compared to the NSE group, and further research should establish the individual dosage of exercise and identify individuals in need of a supervised NSE program or other interventions. Further research is also needed to clarify the pathophysiological mechanisms underlying WAD, improve diagnostics, and plan treatment for individuals who do not improve with the NSEs.

### Conclusion and Clinical Implications

Both the NSEIT and NSE groups demonstrated sustained clinically important changes in disability and pain for approximately 50% of participants. The results showed that NSEIT was not worse than NSE and could be used as a treatment in patients with chronic WAD grades II and III. The results add to previous studies, offering a new way to deliver an NSE program, with important reductions in visits to health care facilities and important symptom reductions for patients with chronic pain and disability after whiplash injuries. No severe adverse events were reported in this study, but participants in both groups experienced mainly temporary (<2 weeks) side effects. The results indicate the importance of knowledge of self-regulating exercises for patients and the importance of physiotherapists having knowledge of how to individualize the progression of exercises. The physiotherapists need to be aware of increased symptoms and slower exercise progression in some individuals with chronic WAD.

## Appendix

Multimedia Appendix 1 Information about the neck-specific exercise program.

Multimedia Appendix 2 The neck-specific exercise program, including exercises to find the deep neck muscles and progression.

Multimedia Appendix 3 Per protocol analyses, within-group differences, self-rated recovery, and adverse events.

Multimedia Appendix 4 CONSORT-eHEALTH checklist (V 1.6.1).

## Abbreviations

GRS: Global Rating Scale
NDI: Neck Disability Index
NSE: neck-specific exercise
NSEIT: neck-specific exercises with internet support
PSFS: Patient-Specific Functional Scale
RCT: randomized controlled trial
VAS: Visual Analog Scale
WAD: whiplash-associated disorder
WDQ: Whiplash Disability Questionnaire

## Data Availability

The data sets generated during this study are available from the corresponding author on reasonable request.
