# Supplementary material for: Efficacy of Neck-Specific Exercise With Internet Support Versus Neck-Specific Exercise at a Physiotherapy Clinic in Chronic Whiplash-Associated Disorders: Multicenter Randomized Controlled Noninferiority Trial
Source: J Med Internet Res. 2023 Jun 20;25:e43888. doi: 10.2196/43888 (PMC10337460; doi:10.2196/43888)
Supplement: Multimedia Appendix 1 [file jmir_v25i1e43888_app1.pdf]

APPENDIX 1

Neck-specific exercise programme

All participants received the same information and neck-specific exercise programme, but it was delivered in two different ways.
The NSEIT group had four visits with the physiotherapist. Information and the exercise programme were delivered via the internet.
The NSE group exercised mainly at the physiotherapy clinic twice a week for 3 months (12 weeks), but with some additional home exercises. The participants in the NSE group received their information when visiting the physiotherapist.

Neck-specific exercises with internet support (NSEIT)

Week 1

- The physiotherapist performed a clinical examination and, based on their analysis, customized an exercise programme from a well-defined set of exercises with a standardized, structured progression plan (Appendix 2). Participants were instructed to read the information on the website, including information regarding the whiplash injury mechanism and the relevant musculoskeletal anatomy and function. The purpose was to explain the mechanism of injury and justify the exercise programme in order to enhance the participants' motivation to perform the exercises. Posture exercises and neck-specific exercises (Appendix 2) designed to facilitate use of the deep neck muscles were introduced.

Week 2

- The physiotherapist followed up on the exercises performed by the participant and asked if they had any questions regarding the information. The aim was to ensure that the participant understood the information correctly and to re-explain it if they did not. The participant was encouraged to read about pain processes, including the neurophysiological and neurobiological processes underlying chronic pain. The aim

was to explain why persistent pain can occur even after the potential injuries to muscles and/or ligaments have healed after the whiplash accident. Another aim was to increase the patients' understanding of the importance of exercising the neck muscles and having good neck posture in order to decrease or prevent pain. Isometric neck-specific exercises in lying and sitting positions were introduced, as well as gym exercises if they were tolerated.

Weeks 3 and 4

The physiotherapist evaluated whether the exercises had been correctly understood and asked if there were any further questions regarding the information on the website. New exercises were introduced and existing exercises progressed, or adjusted to a lower level of the exercise programme if needed (Appendix 2).

Week 7

The physiotherapist evaluated whether the exercises had been correctly understood and asked if there were any further questions regarding the information on the website. The physiotherapist reminded the participants to read about neck pain relapse on the website. New exercises were introduced and existing exercises progressed, or adjusted to a lower level of the exercise programme if needed (Appendix 2).

Technicians were available to assist the participants if any technical difficulties arose regarding the web-based programme.

Neck-specific exercises at a physiotherapy clinic (NSE)

Week 1

- The physiotherapist performed a clinical examination and, based on their analysis, customized an exercise programme from a well-defined set of exercises with a standardized, structured progression plan (Appendix 2). Participants were educated regarding the whiplash injury mechanism and the relevant musculoskeletal anatomy and function. The purpose was to explain the mechanism of injury and justify the exercise programme in order to enhance the participants' motivation to perform the exercises. Posture exercises and neck-specific exercises (Appendix 2) designed to facilitate use of the deep neck muscles were introduced.

Week 2

- The participant was informed and educated about the neurophysiological and neurobiological processes underlying chronic pain. The aim was to explain why persistent pain can occur even after the potential injuries in muscles and/or ligaments have healed after the whiplash accident. Another aim was to increase participants' understanding of the importance of exercising the neck muscles and to have good neck posture in order to decrease or prevent pain. Isometric neck-specific exercises in lying and sitting positions were introduced, as well as gym exercises if they were tolerated.

Weeks 3–9

The physiotherapist evaluated and followed up on the exercise programme and symptoms during the participant's visit to the clinic. The exercises were progressed or, if needed, adjusted to a lower level in the exercise programme (Appendix 2). The participant continued to exercise twice a week at the physiotherapy clinic with additional home exercises according to the exercise programme.

Weeks 10–12

- Information was given to the participant about the risk of neck pain relapse and the physiotherapist discussed strategies for dealing with such a relapse. The aim was to inform participants that neck pain relapse is common and to have a recovery plan. The participants continued training with gradual progression. The physiotherapist and participants discussed where and how the exercise should continue outside the healthcare setting after week 12.
