# Supplementary material for: Efficacy of Neck-Specific Exercise With Internet Support Versus Neck-Specific Exercise at a Physiotherapy Clinic in Chronic Whiplash-Associated Disorders: Multicenter Randomized Controlled Noninferiority Trial
Source: J Med Internet Res. 2023 Jun 20;25:e43888. doi: 10.2196/43888 (PMC10337460; doi:10.2196/43888)
Supplement: Multimedia Appendix 2 [file jmir_v25i1e43888_app2.pdf]

APPENDIX 2
The images are used with the participant's consent.

NECK-SPECIFIC EXERCISES PART 1

Posture exercise

The participant was encouraged to perform a posture exercise every waking hour during the first week. This posture exercise was performed in a sitting position with the instruction to hold the upright position for 10 seconds, rest for a few seconds, then repeat 10 times. The participants were informed that the aim of the exercise was to minimize any unfavourable load on the neck that can increase neck pain, and to find a good neck position for further exercises.

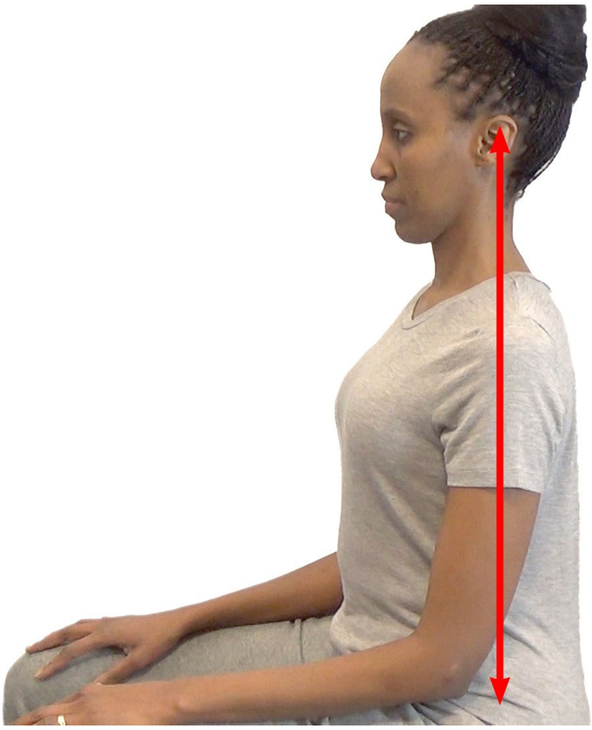

Fig. 1 Posture exercise

Part 1. Exercises to find the deep neck muscles

The neck-specific exercises were performed in a supine position. A folded towel was used to find a good neck posture during the exercises. During these exercises, the aim was to facilitate deep neck muscle activity, and the participant was informed how to minimize contraction of the superficial neck muscles (i.e., m. trapezius, m. sternocleidomastoid, or m. scalene). Achieving a pattern of correct movement was crucial before the patient could begin neck-specific exercises part 2.

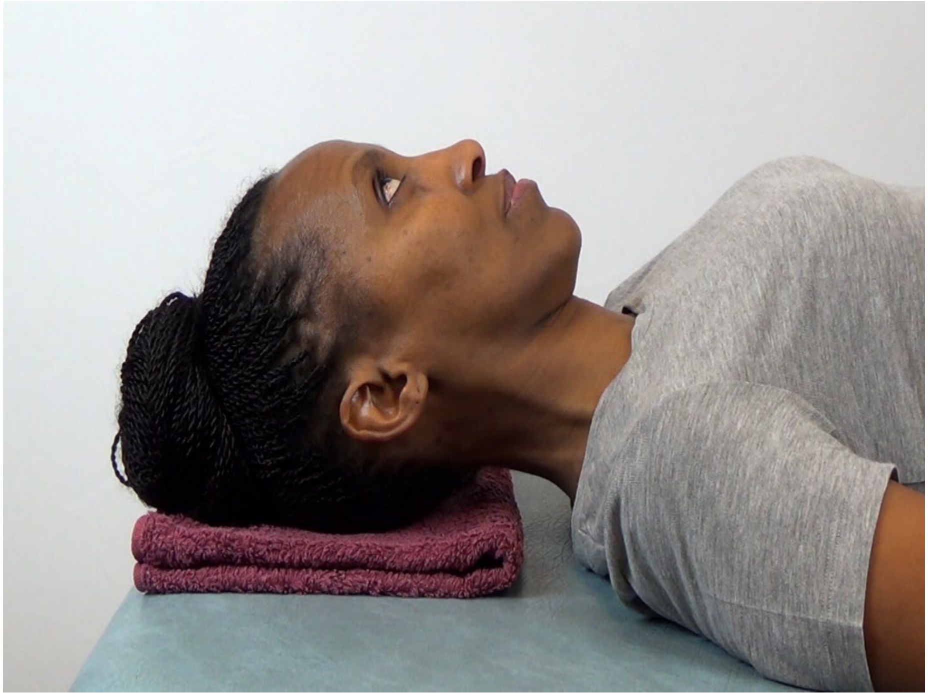

Fig. 2 Extension.

The participants imagined that they were performing a neck extension without moving their head. To facilitate dorsal neck muscle activation, the participants followed the imagined extension with their eyes, looking up and back. The superficial muscles were relaxed during the exercise. The position was held for 3 to 5 seconds, and the exercise was repeated five times.

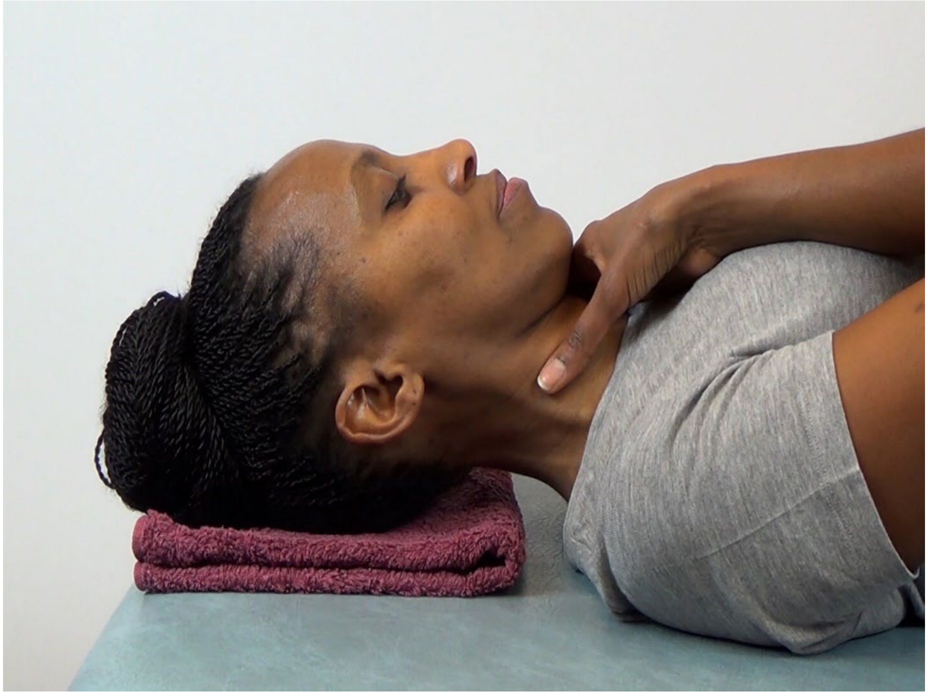

Fig. 3 Flexion.

The starting position was a relaxed jaw with the lips together but teeth apart and a relaxed tongue. The participant imagined that they were performing a gentle nod without moving the head, and with the eyes looking down towards the chest. The participant placed one hand gently on the ventral neck muscles to be aware if the superficial muscles were activated. The superficial muscles were relaxed during the exercise. The position was held for 3 to 5 seconds, and the exercise was repeated five times.

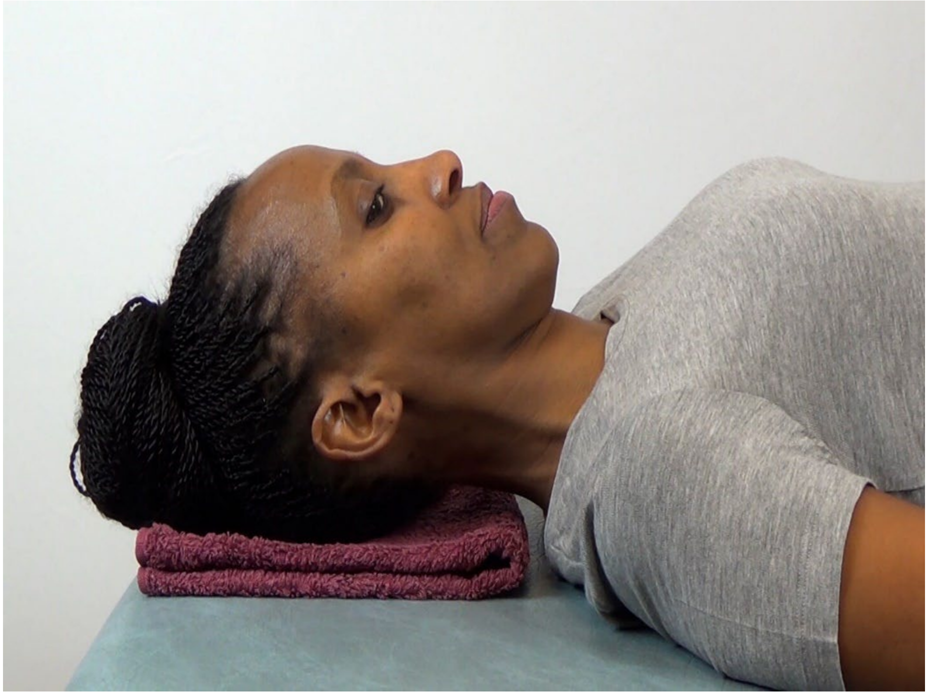

Fig. 4 Rotation.

The participant imagined that their head rotated to the right without moving their head. The participant was told to look to the right by only moving their eyes. The position was held 3 to 5 seconds before going back to neutral position. The superficial muscles were relaxed during the exercise. This exercise was repeated five times. Thereafter, the exercise was performed to the left.

NECK-SPECIFIC EXERCISES PART 2

In part 2, the neck-specific exercises were performed in a supine position with gentle isometric resistance. It was important that the exercises did not provoke radiating pain in the arms and that neck pain did not increase after the exercise programme. A folded towel was used to find a good neck posture during the exercises. During these exercises, the aim was to facilitate deep neck muscle activity against a slight load. The participant was informed about how to minimize contraction of the superficial neck muscles (i.e., m. trapezius, m. sternocleidomastoid, or m. scalene). Achieving a correct movement pattern was crucial before the patient started neck-specific exercises in a sitting position (part 3).

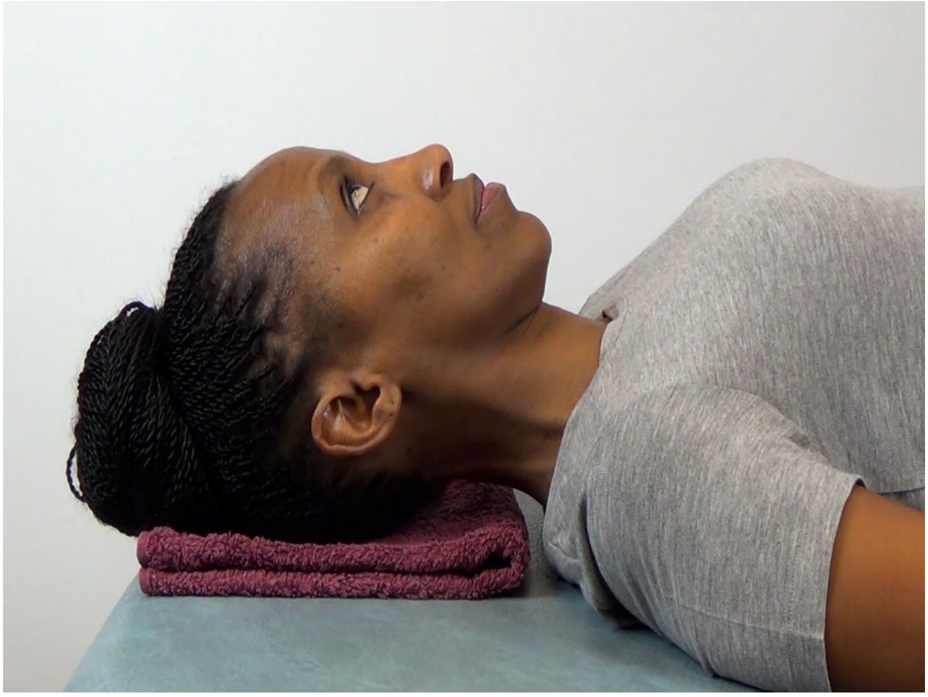

Fig. 5 Extension.

The participant performed a slight neck extension, and they gently pushed their head against the table. Their eyes followed the extension up and back. The superficial muscles were relaxed during the exercise. The position was held for 3 to 5 seconds. The exercise was repeated five times, with successive individualized progression up to 3 x 10 repetitions.

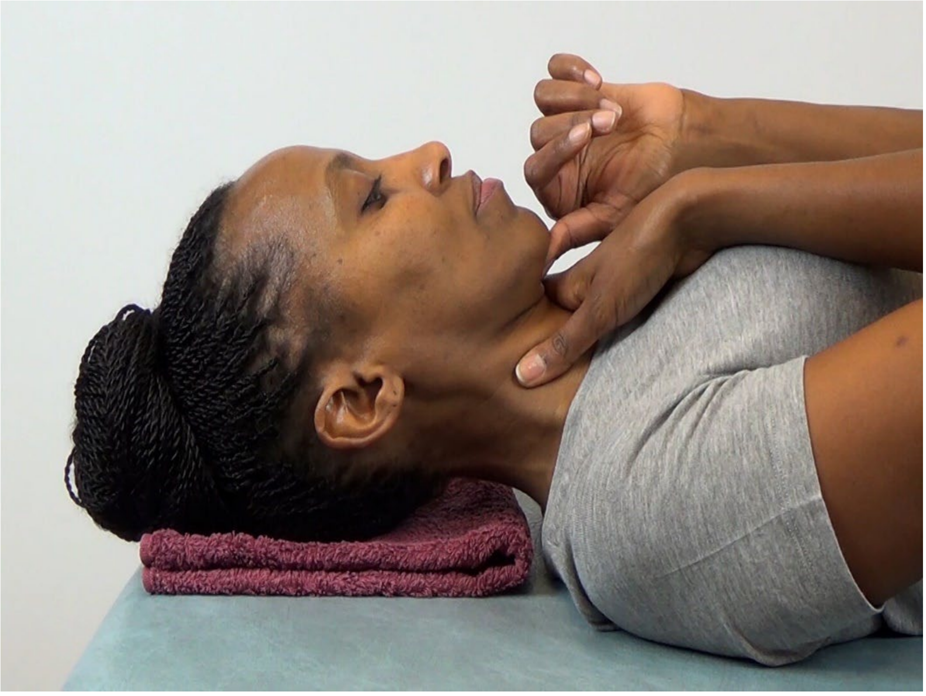

Fig. 6 Flexion.

The participant positioned a hand under their chin as resistance. The participant performed a gentle nod with their eyes looking down towards their chest. The superficial muscles were relaxed during the exercise. The participant placed one hand gently on the superficial ventral neck muscles to be aware if the superficial muscles were activated. The position was held for 3 to 5 seconds. The exercise was repeated five times, with successive individualized progression up to 3 x 10 repetitions.

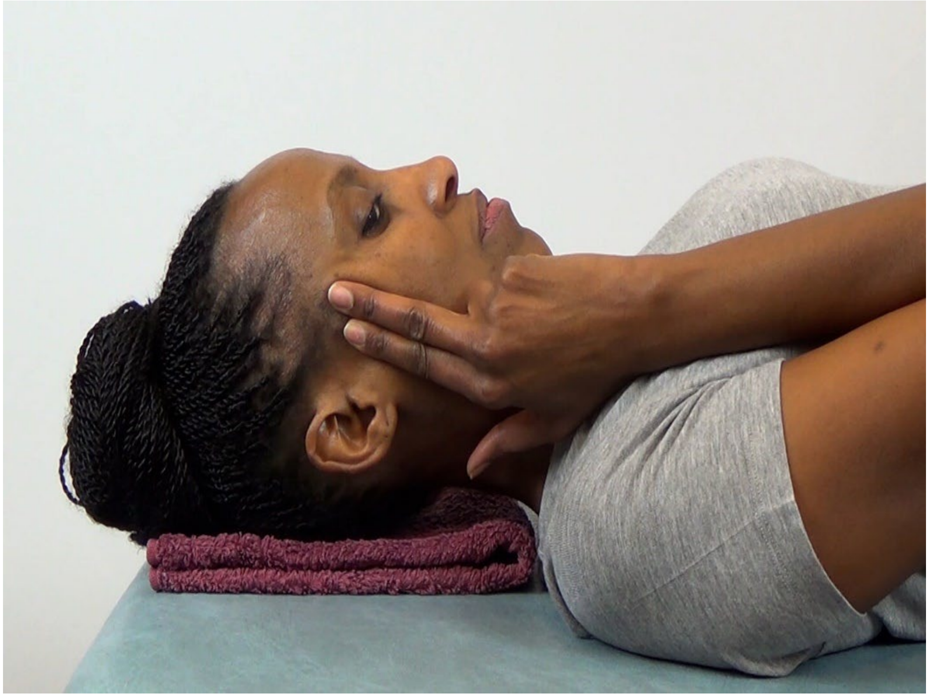

Fig. 7 Rotation.

The participant looked to the left and held two fingers to their right cheekbone (os zygomaticum). They then gently pressed their cheek in a rotational direction to the left. The position was held for 3 to 5 seconds, and the exercise was repeated 5 times, with successive individualized progression up to 3 x 10 repetitions. The superficial muscles were relaxed during the exercise.

NECK-SPECIFIC EXERCISES PART 3

In part 3, the neck-specific exercises were performed in an upright position, sitting on a chair, with feet on the ground. It was important that the exercises did not provoke radiating pain in the arms, and that neck pain did not increase after the exercise programme beyond muscle soreness. The patients were instructed to maintain good neck posture during the exercises. The neck position was a slight upper cervical flexion nod when performing isometric training of the neck muscles in flexion, extension, and rotation. Achieving a correct movement pattern was crucial before the patient started neck-specific exercises in part 4.

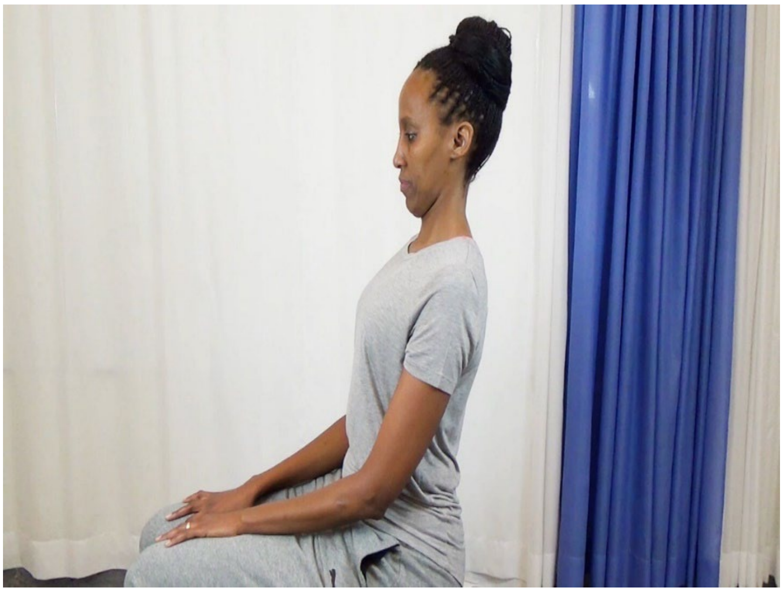

Fig. 8 Extension.

The participant leaned their body back from the pelvis with their neck in line with their torso. The leaning position was held for 3 to 5 seconds, going back to neutral position and repeating 5 times, with successive individualized progression up to 3 x 10 repetitions.

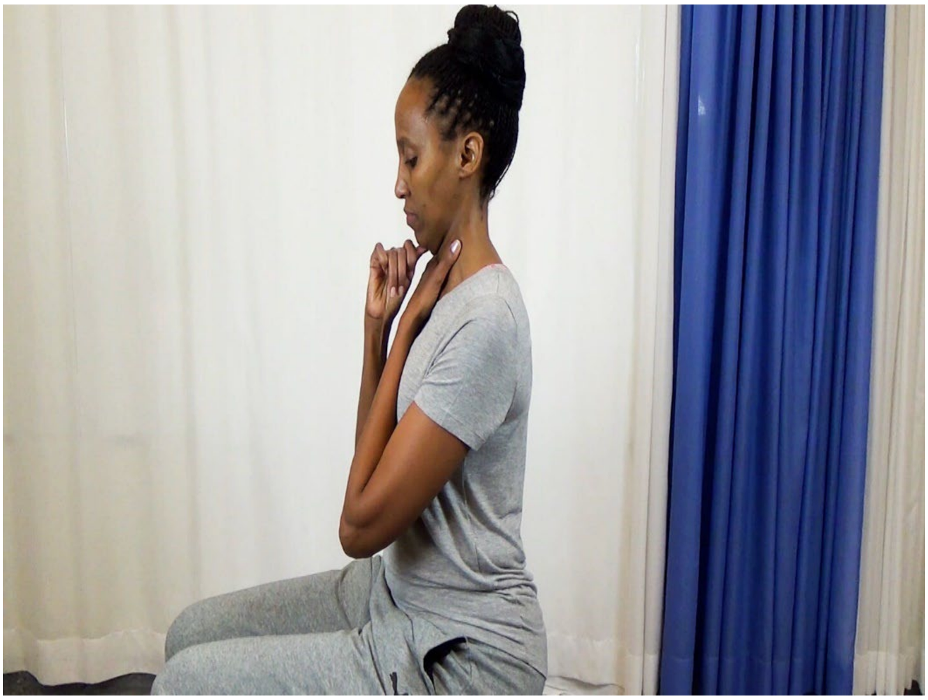

Fig. 9 Flexion.

The exercise position was upright, with the participant sitting on a chair with their feet on the ground. The participant made a slight nod with the upper cervical spine, looking down the chest and holding a thumb under their chin as resistance. They gently pressed their chin onto the thumb, resulting in isometric contraction. The superficial muscles were relaxed during the exercise. The contraction was held for 5 seconds and the exercise repeated 5 times, with successive individualized progression up to 3 x 10 repetitions.

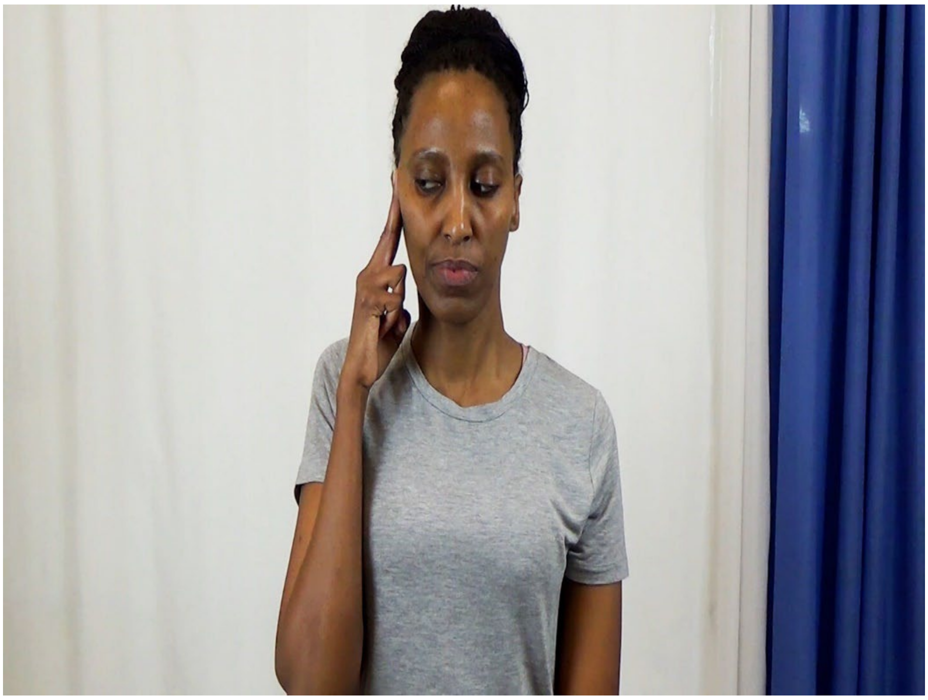

Fig. 10 Rotation.

The participant looked to the left and held two fingers to their right cheekbone (os zygomaticum). They then gently pushed their cheek in a rotational direction to the left. The superficial muscles were relaxed during the exercise. The contraction was held for 5 seconds and the exercise repeated 5 times, with successive individualized progression up to 3 x 10 repetitions.

NECK-SPECIFIC EXERCISES PART 4

In part 4, the neck-specific exercises in extension, flexion, and lateral flexion were performed in a sitting position with the participant's feet on the ground. A rubber band or gym exercises, or a combination of the two, was chosen.

Rubber band exercises

A flat rubber band with light resistance was placed around the head at the height of the forehead/eyes. All participants had a rubber band of their own to ensure good hygiene and for home exercises. The participant was instructed to maintain good neck posture during the exercises. The neck position was slight upper cervical flexion and retraction when performing training of the neck muscles in flexion, extension, lateral flexion, and rotation. The focus of the exercise was low-load endurance training. One exercise in each direction was chosen. The exercises were progressed for some participants using a rubber band with more resistance.

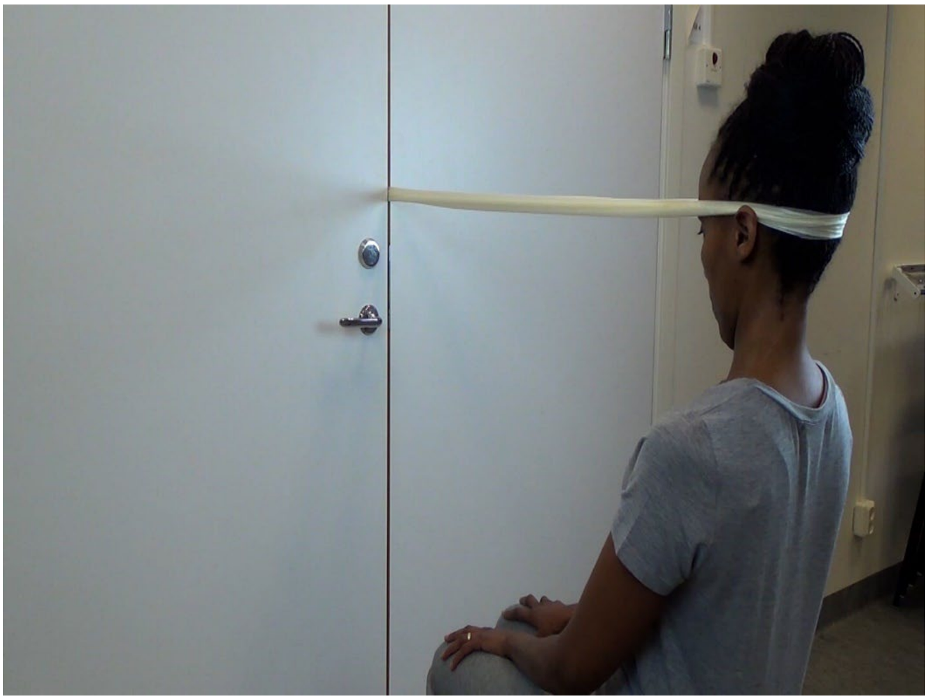

Fig. 11 Extension.

The participant leaned their body backward from the pelvis with their neck in line with their torso. The participant repeated the exercise five times, with successive individualized progression up to 3 x 30 repetitions.

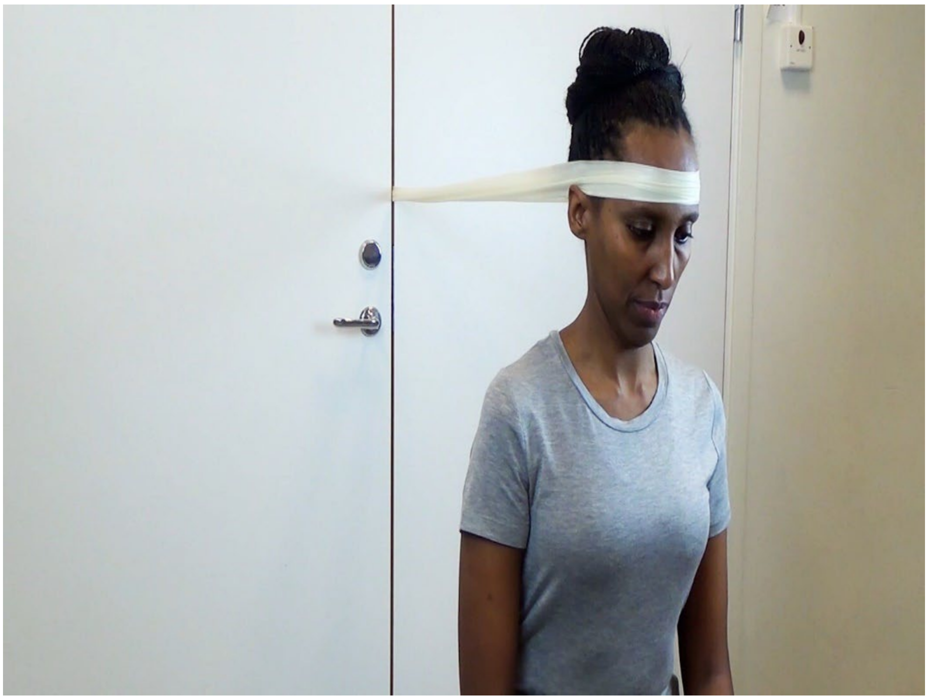

Fig. 12 Flexion.

The participant leaned their body forward from the pelvis with their neck in line with their torso. The exercise was repeated five times, with successive individualized progression up to 3 x 30 repetitions.

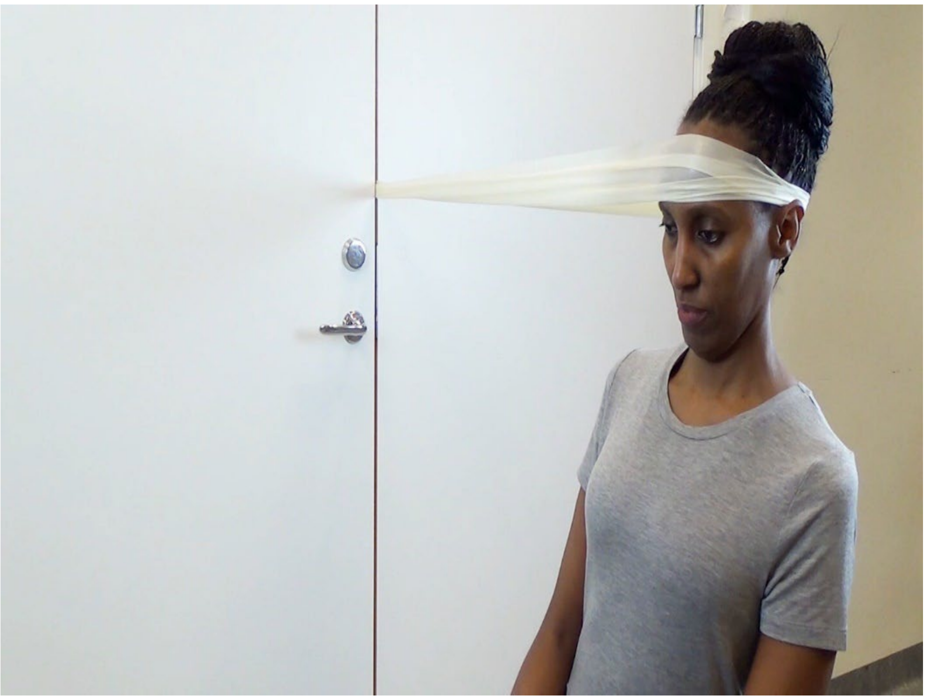

Fig. 13 Lateral flexion.

The participant leaned their body to the left from the pelvis with their neck in line with the torso. The contraction was held for 3 seconds and repeated 5 times, with successive individualized progression up to 3 x 30 repetitions. The exercise was then performed to the right.

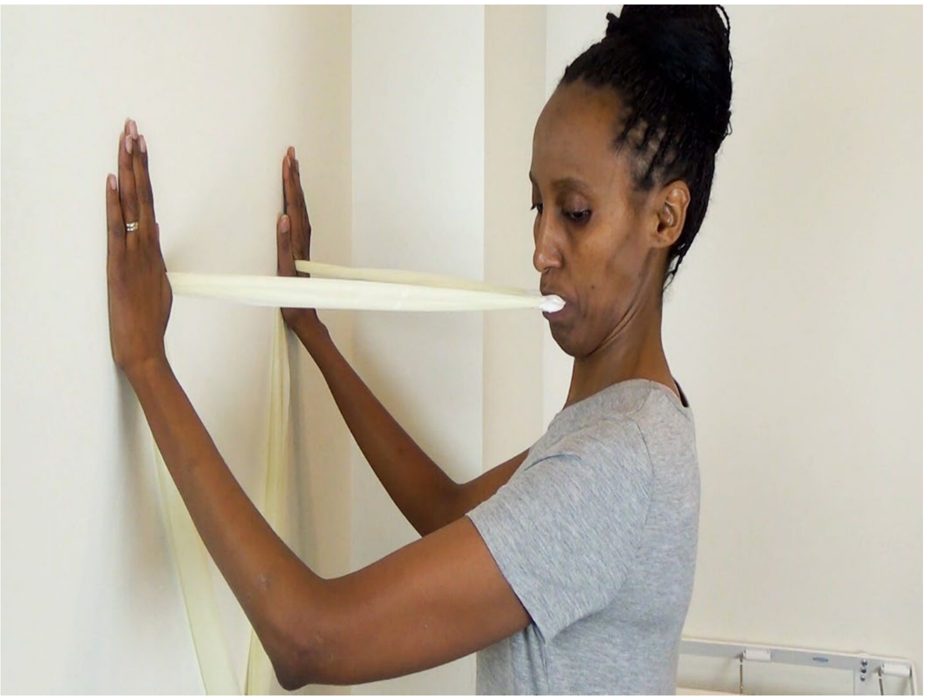

Fig. 14 Rotation.

The exercise was performed in an upright standing position using a rubber band with light resistance. The participant held the rubber band gently between their teeth with their hands in line with their mouth. Most of the participants preferred to have a thin cloth between their mouth and the rubber band. The neck was rotated approximately 30 degrees to the left with good neck posture. The neck was then rotated to the right. The exercise was repeated five times. This exercise successively progressed in terms of degrees of neck rotation and up to 3 x 30 repetitions.

NECK-SPECIFIC EXERCISES PART 4. GYM EXERCISES

Gym exercises were performed by the neck-specific exercise group at the physiotherapy clinic. Gym exercises could also be performed at a private gym outside the healthcare setting by participants in the group with internet support, in addition to their home exercises.

During the gym exercises, the participants were instructed to maintain a correct neck posture of slight upper cervical flexion and retraction when isometric training of the neck muscles in flexion, extension, and lateral flexion was performed. The starting load (weighted pulley) was generally lower for women (0.5 kg) than for men (1 kg). The focus of the exercise was neck muscle endurance training, starting with 5–10 repetitions and gradually progressing up to 3 sets of 30 repetitions. The physiotherapist chose one of two exercises (pulley or guild board) for each direction: extension, flexion, and lateral flexion exercises. The rotation exercise was dynamic, using the pulley and/or an elastic rubber band that was gently held between the teeth (figure 14) or in the supine position with load.

The exercises should not provoke neck pain or other symptoms, such as radiating sensations, paraesthesia, nausea, etc. Temporary muscle soreness was permitted. To prevent pain provocation, it was very important to maintain correct posture during the exercises, and the posture was adjusted if needed.

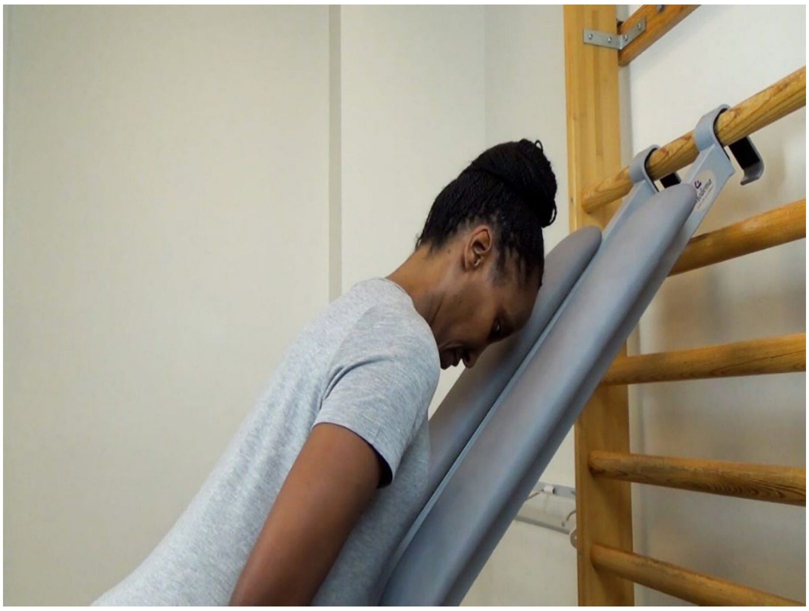

Fig. 15 Extension.

The guild board was placed at an appropriate height on the wall bar. Lower attachment is more strenuous. A small nod of the upper cervical spine was performed and the chin slightly retracted. The participants leaned backwards from the pelvis with their torso, neck, and head in line. The exercise was repeated five times, with successive individualized progression up to 3 x 30 repetitions.

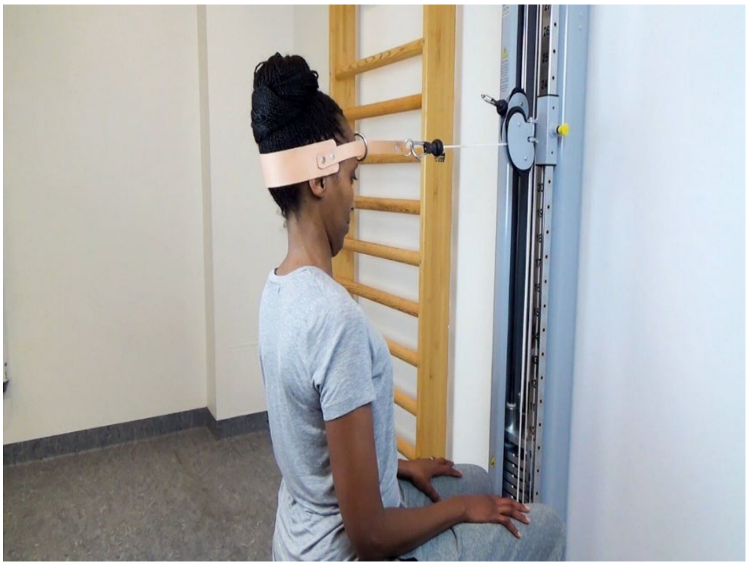

Fig. 16 Extension.

The participants leaned backwards from the pelvis with their neck in line with the torso. The exercise was repeated five times, with successive individualized progression up to 3 x 30 repetitions.

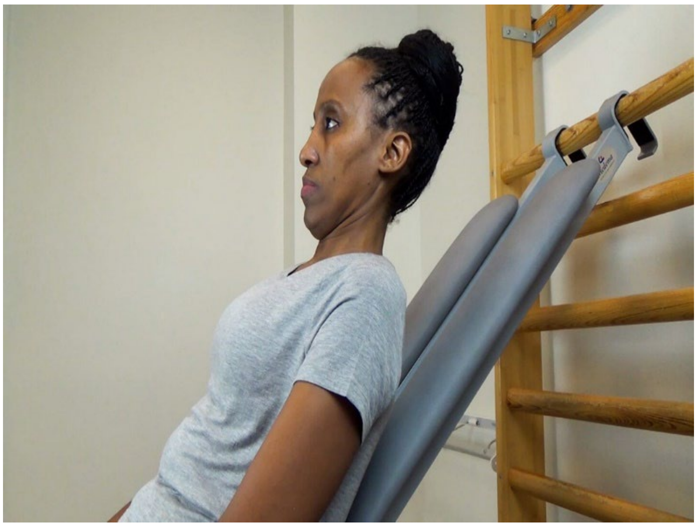

Fig. 17 Flexion.

The guild board body was placed at an appropriate height. Lower attachment is more strenuous. The upper cervical spine was slightly flexed in a nod and the chin slightly retracted. The participants leaned slightly forward from the pelvis, lifting their head in line with the torso. The exercise was repeated five times, with successive individualized progression up to 3 x 30 repetitions.

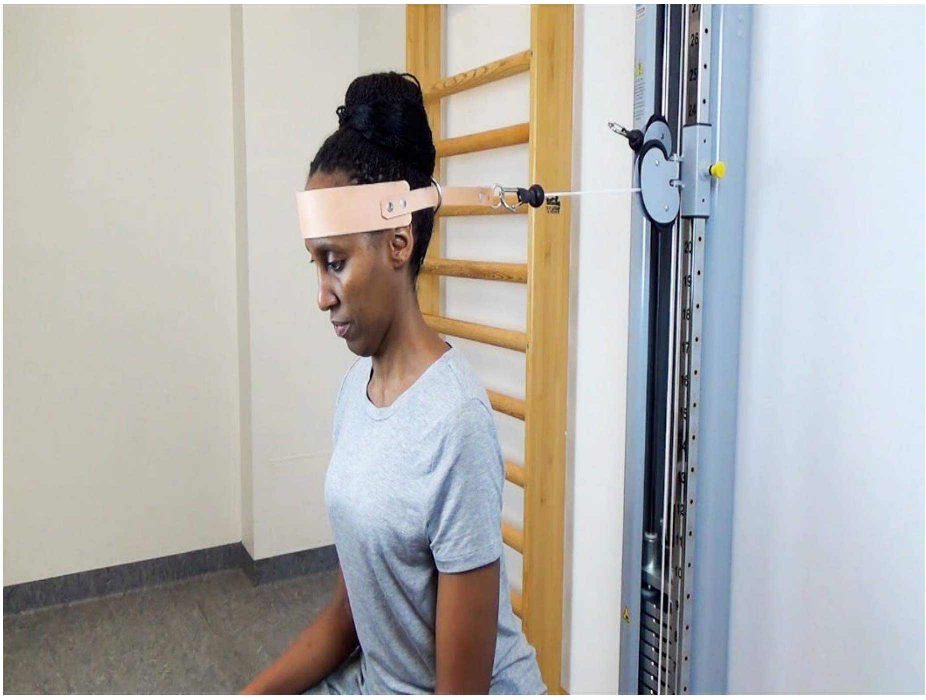

Fig. 18 Flexion.

The participants leaned forwards from the pelvis with their neck in line with the torso. The starting position was with a slight upper cervical spine nod and a slightly retracted neck. The

exercise was repeated five times, with successive individualized progression up to 3 x 30 repetitions.

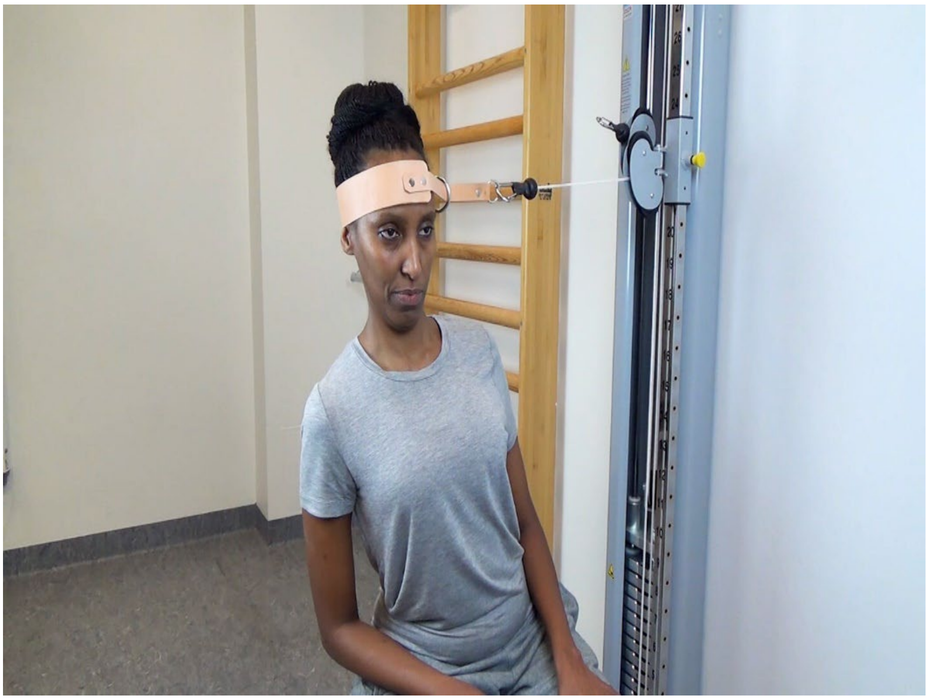

Fig. 19 Lateral flexion.

The participants leaned sideways to the left from the pelvis, with their neck in line with the torso. The exercise was repeated five times, with successive individualized progression up to 3 x 30 repetitions. The exercise was then performed to the right.

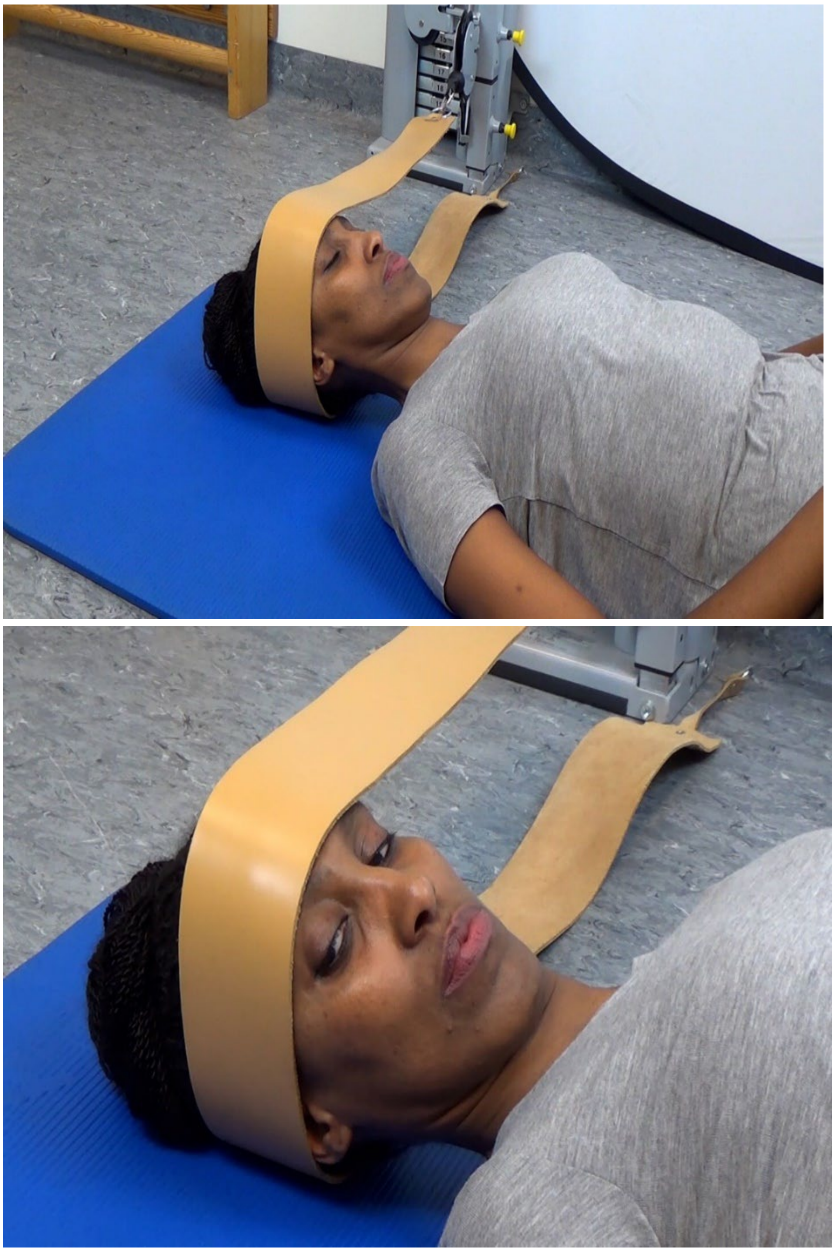

Fig. 20 Rotation against resistance from a leather band.

The patient lay on a mat on the floor with the leather band tightly placed around their head at the height of the temple. The neck was rotated approximately 30–50 degrees to the left with good neck posture. The exercise was repeated five times. The neck was then rotated to the right. The exercise was successively progressed in terms of degrees of neck rotation and up to 3 x 30 repetitions.

ADDITIONAL SHOULDER AND CORE EXERCISES

Additional exercises were designed to improve posture, with strength and endurance exercises for the shoulder and core muscles. When the participants started to exercise in parts 2 and 3, a recommendation was made to add exercises for the shoulders (figure 20, 21) and the back (figure 22). These exercises were repeated three times a week. Approximately 2–4 weeks later, the next shoulder exercise (figure 22) and exercises for the ventral neck muscles (figure 23, 24) were added. After week 7 in the exercise programme, an abdominal muscle exercise (figure 24) was added. Temporary muscle soreness was permitted but no provocation of arm pain or other neurological symptoms.

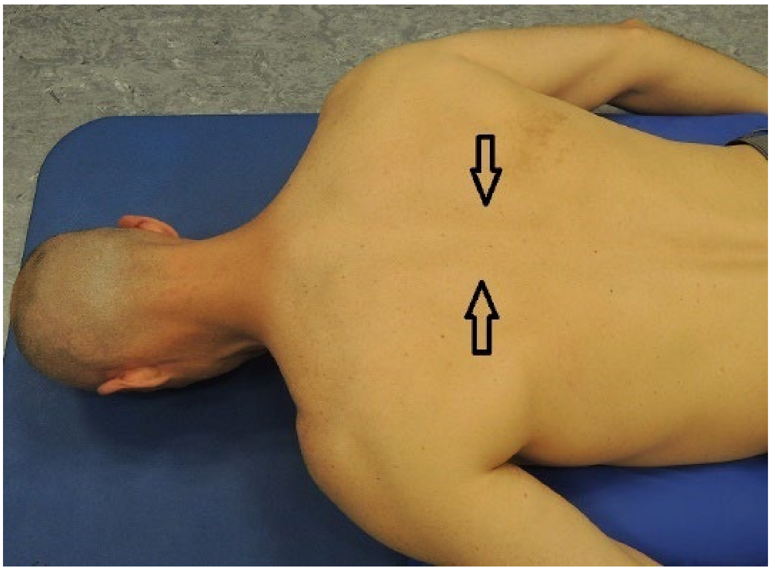

Fig. 21 Shoulder muscle activation.

The exercise was performed prone, with forehead against the floor. The arms were relaxed and resting against the floor. The shoulder blades were gently pressed together without muscle activation in the upper trapezius or arm muscles. The exercises started with 3 sets of 5 repetitions and progressively increased towards 3 sets of 10 repetitions.

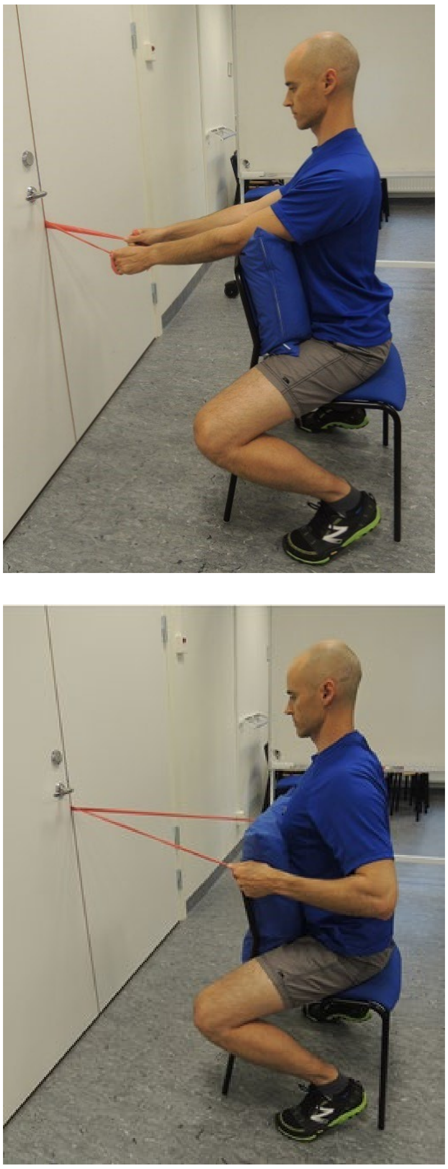

Fig. 22 Shoulder exercise.

The exercise position was upright, sitting on a chair with feet on the ground. A rubber band was fixed in line with the arms, with the elbow flexed at 90º. The rubber band was stretched with extended arms in front of the body in line with the shoulders, and participant retracted their arms as though rowing a boat. The exercises started with 3 sets of 5 repetitions and progressively increased towards 3 sets of 10 repetitions.

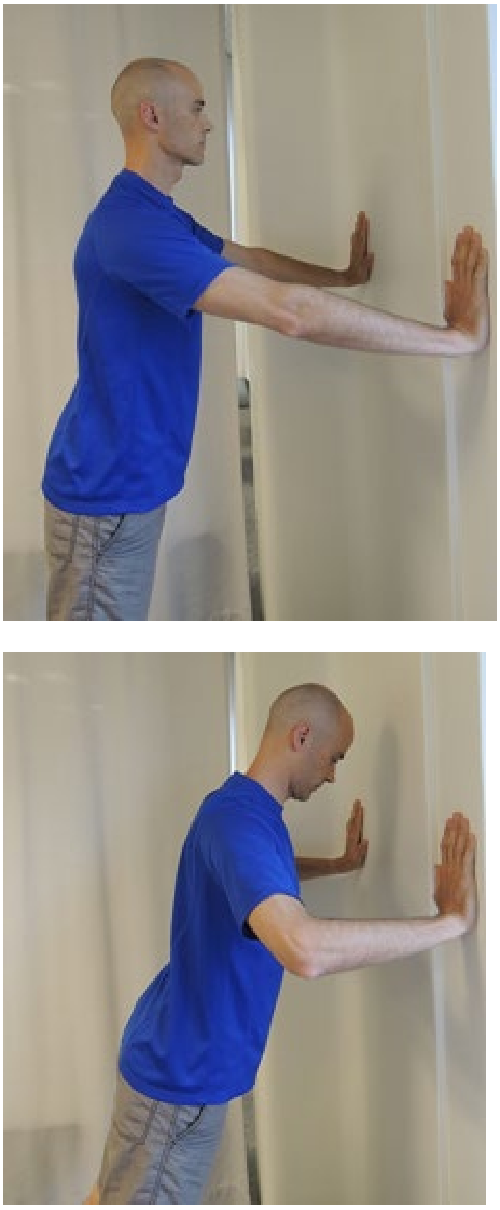

Fig. 23 Shoulder exercise.

The exercise position was standing, with the participant’s hands on the wall in line with the shoulders. The neck position was in line with the torso, and the neck posture was a slight upper cervical flexion and retraction during the exercise. The exercise started with 3 sets of 5 repetitions of the push-ups and progressively increased towards 3 sets of 10 repetitions.

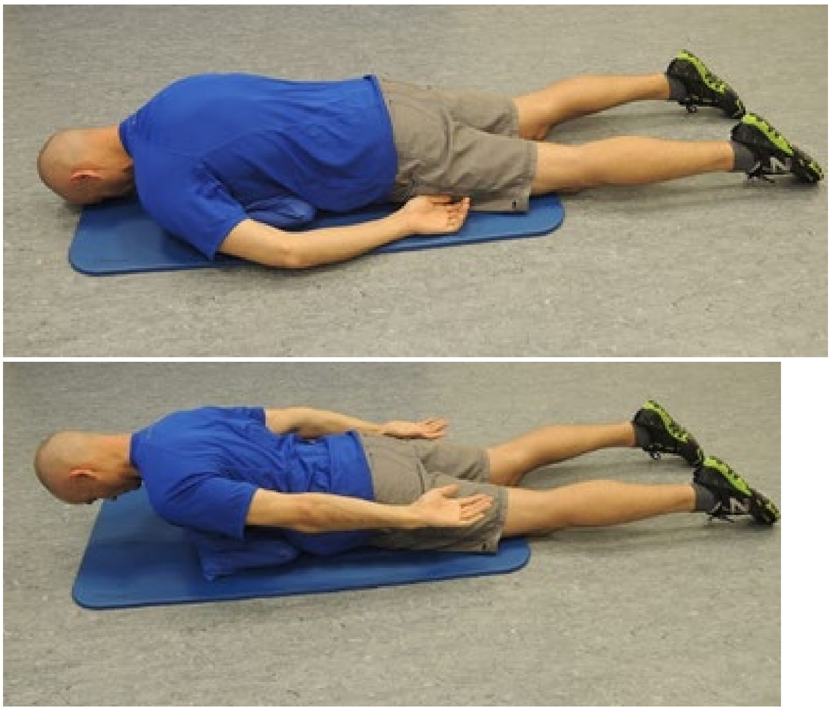

Fig. 24 Back exercise.

The exercise was performed prone, with a pillow under the chest. The neck position was a gentle nod and retracted chin during the exercise, and the neck was in line with the torso. The participant lifted their upper body approximately 10 cm from the floor, with the upper body in a straight, not over-extended position. The exercise started with 3 sets of 5 repetitions and was progressively increased towards 3 sets of 10 repetitions.

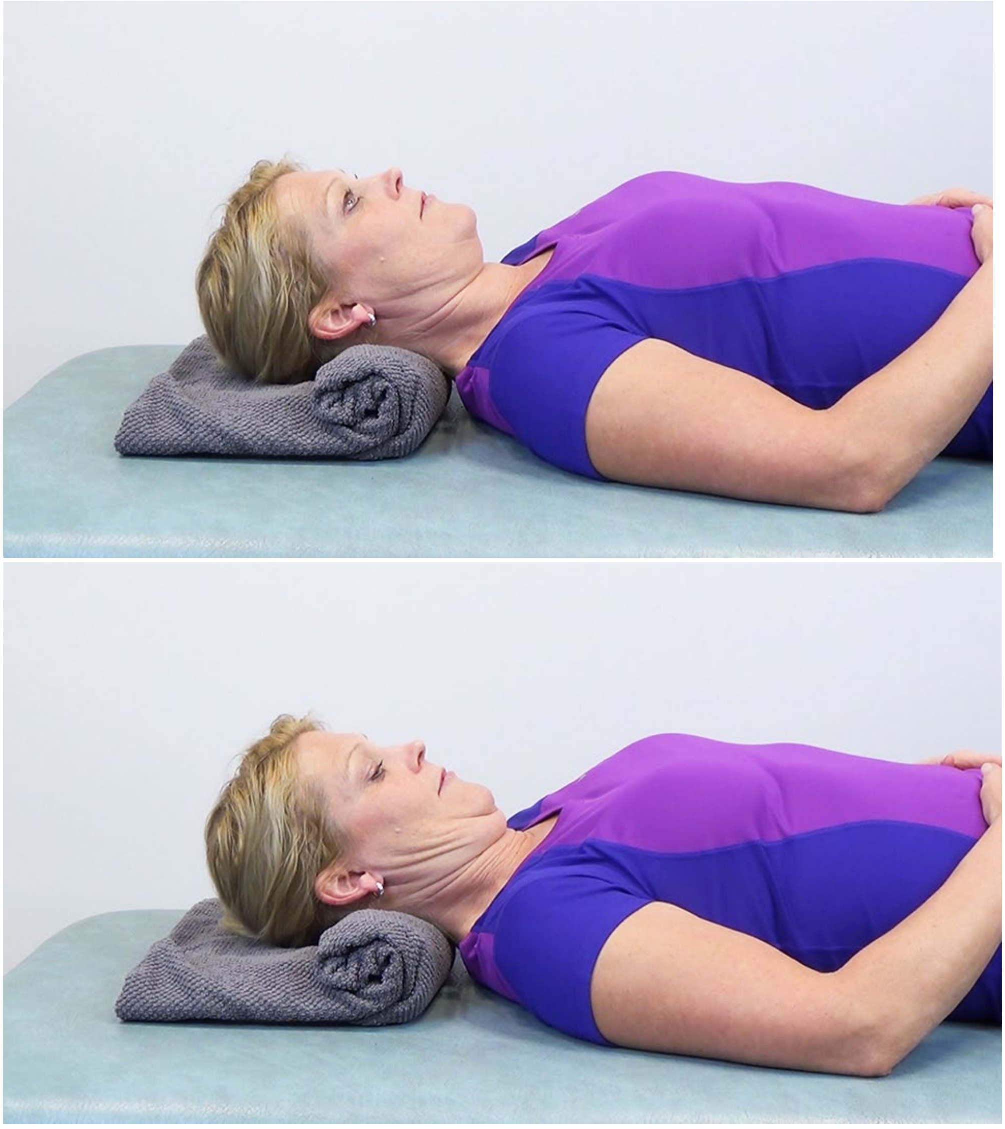

Fig. 25 Ventral muscle exercise.

The exercise was performed supine. A folded towel was rolled and filled the cervical lordosis without changing the good neck posture. The participant looked down and retracted their chin, performing a slight nod without lifting their head, and held the position for 3 seconds. The exercise was repeated 5 to 10 times.

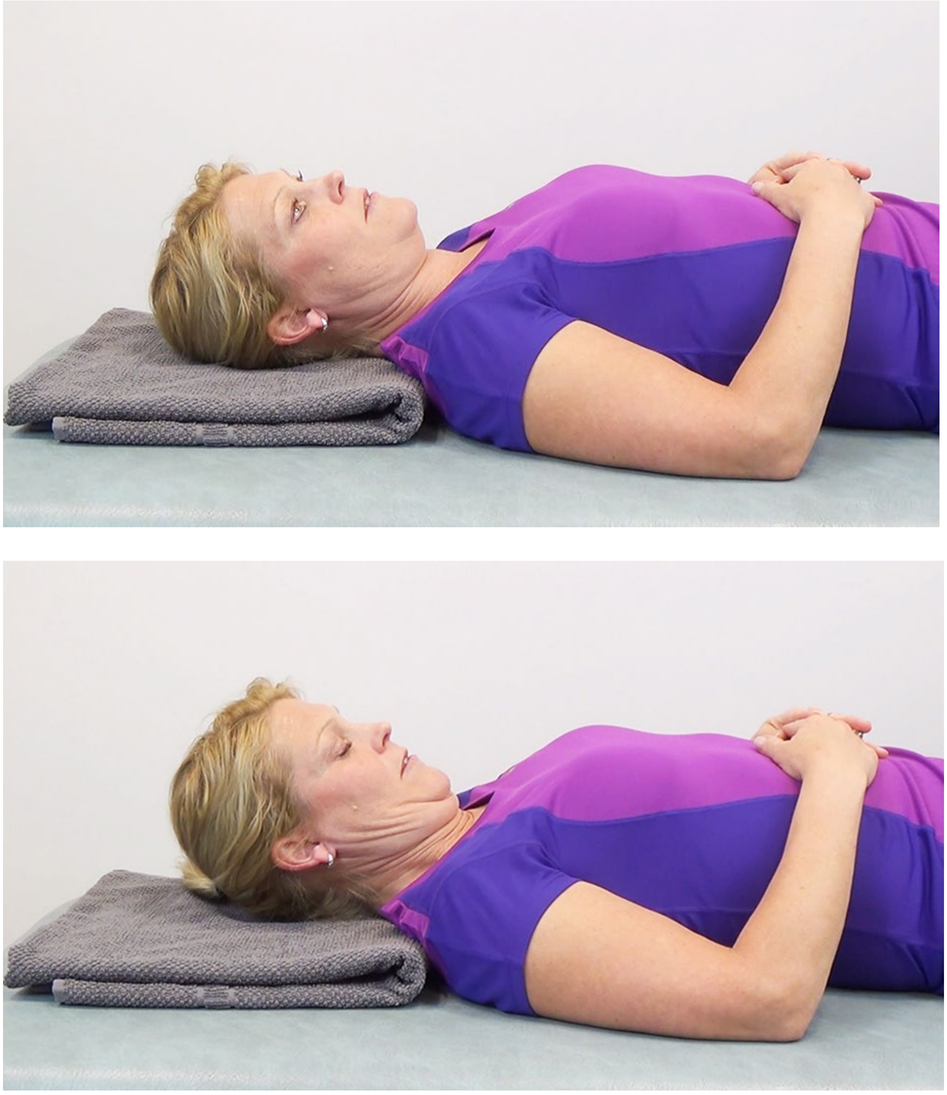

Fig. 26 Ventral neck muscle exercise.

The exercise was performed supine. A folded towel was used to find a good neck posture during the exercise. The participant performed a slight nod of the upper cervical spine to hold the chin in, and then lifted their head slightly above the towel, approximately 3 cm. The participants held the contraction for 15 seconds, repeating it twice if possible. The exercise should not provoke neck pain or other symptoms, such as radiating sensations, paraesthesia, nausea, etc. To prevent pain provocation, it was very important to maintain the chin retraction during the exercise. If it was not possible for the participant to maintain the position, the exercise was stopped. The exercise was progressively increased to a holding time of 30 to 60 seconds.

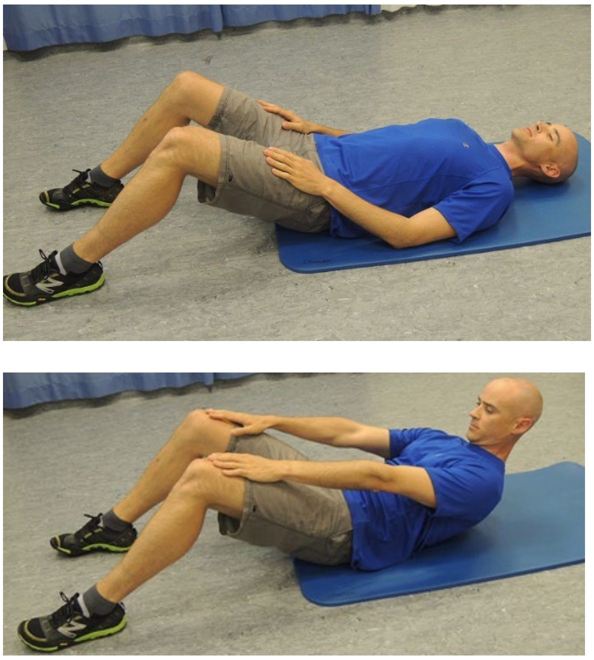

Fig. 27 Sit ups.

The exercise was performed supine, with knees bent. The participants performed a slight nod of the upper cervical spine and held their chin in. They placed their hands on their legs, with their back pushed to the floor. They then raised their body until their hands touched their knees. When returning to a lying position, the position of the chin was held. The exercise started with 3 sets of 5 repetitions and was progressively increased towards 3 sets of 10 repetitions.
