# Supplementary material for: Efficacy of Neck-Specific Exercise With Internet Support Versus Neck-Specific Exercise at a Physiotherapy Clinic in Chronic Whiplash-Associated Disorders: Multicenter Randomized Controlled Noninferiority Trial
Source: J Med Internet Res. 2023 Jun 20;25:e43888. doi: 10.2196/43888 (PMC10337460; doi:10.2196/43888)
Supplement: Multimedia Appendix 3 [file jmir_v25i1e43888_app3.docx]

**Appendix 3**

**Per protocol analyses**

Per protocol analyses (participants with compliance with exercise (defined as at least 50% self-reported attendance to exercises) of primary and secondary outcomes. NSEIT (n = 46, 75%) and NSE (n = 59; 94%), Table 1.

| **Table 1.** Per protocol analyses of between-group effects (95% confidence interval) in primary and secondary outcomes at 3 and 15 months. Positive values indicate favourable change in NSEIT ^a^ compared to NSE ^b^ | | | | | |
| --- | --- | --- | --- | --- | --- |
|  | Change at 3 months | | Change at 15 months | | Main effect of Time × Group |
|  | NSEIT – NSE | *P* value | NSEIT – NSE | *P* value | *F*^c^ */ P* value |
| **Primary outcome** |  |  |  |  |  |
| NDI (%) ^d^ | 2.9 (−1.4 to 7.2) | .19 | 2.2 (−2.7 to 7.2) | .36 | 0.89 / .41 |
| **Secondary outcome** |  |  |  |  |  |
| Neck pain average ^e^ | 1.1 (−8.3 to 10.5) | .82 | −4.0 (−13.0 to 5.1) | .39 | 0.85 / .43 |
| Neck pain now ^e^ | −3.3 (−12.2 to 5.6) | .46 | −5.3 (−14.2 to 3.6) | .24 | 0.69 / .50 |
| Arm pain average ^f^ | 5.0 (−3.9 to 13.8) | .27 | −2.7 (−11.9 to 6.5) | .56 | 1.42 / .24 |
| Arm pain now ^f^ | 5.4 (−2.3 to 13.0) | .17 | −2.7 (−10.9 to 5.6) | .53 | 2.26 / .11 |
| WDQ ^g^ | −0.5 (−8.9 to 8.0) | .91 | −1.2 (−9.4 to 7.1) | .77 | 0.43 / .96 |
| PSFS ^h^ Work | 0.2 (−1.0 to 1.3) | .76 | 0.9 (−0.3 to 2.2) | .14 | 1.42 / .25 |
| PSFS ^h^ Leisure time | 0.1 (−1.2 to 1.1) | .90 | 1.0 (−0.4 to 2.4) | .17 | 1.79 / .17 |
| PSFS ^h^ Physical activity | 0.5 (−0.9 to 2.0) | .46 | 1.2 (−0.3 to 2.6) | .12 | 1.27 / .28 |
| EQ-5D-3L index ^i^ | 0.04 (−0.05 to 0.13) | .43 | −0.02 (−0.12 to 0.07) | .65 | 1.29 / .30 |
| EQ VAS ^j^ | −1.9 (−10.8 to 7.1) | .68 | −7.2 (−15.6 to 1.2) | .09 | 1.45 / .24 |
| ^a^NSE; Neck-specific exercise at physiotherapy clinic  ^b^NSEIT; Neck-specific exercise with internet support  ^c^*F*= F value, repeated measures analysis baseline to 3 and 15 months  ^d^NDI; Neck Disability Index, scored from 0% (no disability) to 100% (high disability)  ^e^Neck pain average previous week and now; Visual analogue scale, scored from 0 mm (no pain) to 100 mm (worst imaginable pain)  ^f^Arm pain average previous week and now; Visual analogue scale, scored from 0 mm (no pain) to 100 mm (worst imaginable pain)  ^g^WDQ; Whiplash Disability Questionnaire, scored from 0 (no disability) to 130 (high disability)  ^h^PSFS; Patient-Specific Functional Scale, scored from 0 (unable to do) to 10 (functional level equal to pre-injury status)  ^i^EQ-5D-3L index, scored from −0.59 to 1, where 1 indicating ‘full health’ and 0 ‘a state as bad as being dead’  ^j^EQ VAS; Visual analogue scale, scored from 0 (the worst imaginable health state) to 100 (the best imaginable health state) | | | | | |

**Within-group differences**

Within-group differences in NSEIT and NSE are shown in Table 2.

| **Table 2.** Within-group mean change (95% confidence interval) in primary and secondary outcomes at 3 and 15 months | | | | |
| --- | --- | --- | --- | --- |
|  | **Within-group change NSEIT ^a^** | | | |
|  | 3 months − Baseline | 15 months − Baseline | *F*^c^ */ P* value | Effect size |
| **Primary outcome** |  |  |  |  |
| NDI (%) ^d^ | −9.1 (−12.3 to −6.0) | −10.1 (−13.7 to −6.5) | 26.66 / <.001 | 1.33 |
| **Secondary outcome** |  |  |  |  |
| Neck pain average ^e^ | −13.9 (−20.9 to −6.9) | −15.8 (−22.7 to −8.8) | 14.77 / <.001 | 1.04 |
| Neck pain now ^e^ | −10.0 (−16.7 to −3.2) | −11.5 (−18.3 to −4.8) | 8.56 / <.001 | 0.79 |
| Arm pain average ^f^ | −9.0 (−15.2 to −2.8) | −7.9 (−15.0 to −0.7) | 6.23 / .003 | 0.79 |
| Arm pain now ^f^ | −5.9 (−11.6 to −0.3) | −5.2 (−11.6 to 1.1) | 3.17 / .046 | 0.48 |
| WDQ ^g^ | −9.8 (−15.8 to −3.7) | −15.3 (−21.4 to −9.1) | 16.19 / <.001 | 1.05 |
| PSFS ^h^ Work | 1.9 (1.1 to 2.6) | 2.9 (2.0 to 3.8) | 32.55 / <.001 | 1.40 |
| PSFS ^h^ Leisure time | 2.3 (1.5 to 3.1) | 3.5 (2.5 to 4.5) | 33.44 / <.001 | 1.52 |
| PSFS ^h^ Physical activity | 1.9 (0.9 to 3.0) | 3.8 (2.7 to 4.9) | 28.41 / <.001 | 1.60 |
| EQ-5D-3L index ^i^ | 0.07 (0.005 to 0.13) | 0.08 (0.005 to 0.15) | 3.65 / .029 | 0.43 |
| EQ VAS ^j^ | 6.2 (−0.3 to 12.8) | 5.5 (−0.8 to 11.8) | 3.18 / .045 | 0.45 |
|  | **Within-group change NSE ^b^** | | | |
|  | 3 months − Baseline | 15 months − Baseline | *F*^c^ */ P* value | Effect size |
| **Primary outcome** |  |  |  |  |
| NDI (%) ^d^ | −7.7 (−10.9 to −4.6) | −9.3 (−12.8 to −5.7) | 21.21 / <.001 | 1.19 |
| **Secondary outcome** |  |  |  |  |
| Neck pain average ^e^ | −13.7 (−20.8 to −6.6) | −19.1 (−26.1 to −12.1) | 19.55 / <.001 | 1.19 |
| Neck pain now ^e^ | −14.3 (−21.2 to −7.5) | −17.6 (−24.4 to −10.9) | 19.11 / <.001 | 1.18 |
| Arm pain average ^f^ | −2.9 (−9.2 to 3.4) | −7.4 (−14.6 to −0.2) | 2.75 / .068 | 0.43 |
| Arm pain now ^f^ | 0.7 (−5.1 to 6.4) | −5.6 (−12.0 to −0.8) | 3.01 / .053 | 0.46 |
| WDQ ^g^ | −12.7 (−18.7 to −6.8) | −18.4 (−24.4 to −12.4) | 24.83 / <.001 | 1.31 |
| PSFS ^h^ Work | 1.6 (0.8 to 2.4) | 2.4 (1.5 to 3.3) | 16.92 / <.001 | 1.14 |
| PSFS ^h^ Leisure time | 2.1 (1.3 to 2.9) | 3.0 (2.0 to 4.0) | 25.93 / <.001 | 1.35 |
| PSFS ^h^ Physical activity | 1.2 (0.2 to 2.2) | 2.7 (1.6 to 3.7) | 18.99 / <.001 | 1.12 |
| EQ-5D-3L index ^i^ | 0.03 (−0.04 to 0.09) | 0.07 (−0.002 to 0.14) | 2.61 / .078 | 0.43 |
| EQ VAS ^j^ | 7.5 (1.0 to 14.0) | 10.9 (4.6 to 17.2) | 8.66 / <.001 | 0.74 |
| ^a^NSE; Neck-specific exercise at physiotherapy clinic  ^b^NSEIT; Neck-specific exercise with internet support  ^c^*F*= F value, repeated measures analysis baseline to 3 and 15 months  ^d^NDI; Neck Disability Index, scored from 0% (no disability) to 100% (high disability)  ^e^Neck pain average previous week and now; Visual analogue scale, scored from 0 mm (no pain) to 100 mm (worst imaginable pain)  ^f^Arm pain average previous week and now; Visual analogue scale, scored from 0 mm (no pain) to 100 mm (worst imaginable pain)  ^g^WDQ; Whiplash Disability Questionnaire, scored from 0 (no disability) to 130 (high disability)  ^h^PSFS; Patient-Specific Functional Scale, scored from 0 (unable to do) to 10 (functional level equal to pre-injury status)  ^i^EQ-5D-3L index, scored from −0.59 to 1, where 1 indicating ‘full health’ and 0 ‘a state as bad as being dead’  ^j^EQ VAS; Visual analogue scale, scored from 0 (the worst imaginable health state) to 100 (the best imaginable health state) | | | | |

**Self-rated recovery**

Self-rated recovery was measured with the 11-point Global Rating scale (GRS; −5 = vastly worse, 0 = unchanged, 5 = completely recovered) at 3- and 15-month follow-ups (Table 3). In the NSEIT and NSE groups, 63 to 64 % of the participants reported that they were recovered at the 3-month follow-up and 65 to 69% at the 15-month follow-up (≥ +2).

| **Table 3.** Global Rating Scale at 3- and 15-month follow-up in the NSEIT ^a^ and NSE ^b^ groups | | | | |
| --- | --- | --- | --- | --- |
|  | 3 months | | 15 months | |
|  | NSEIT | NSE | NSEIT | NSE |
| GRS values | n (valid %) | n (valid %) | n (valid %) | n (valid %) |
| −5 vastly worse | 0 | 0 | 0 | 0 |
| −4 | 1 (2%) | 1 (2%) | 0 | 0 |
| −3 | 1 (2%) | 1 (2%) | 2 (4%) | 3 (5%) |
| −2 | 1 (2%) | 3 (5%) | 1 (2%) | 1 (2%) |
| −1 | 2 (3%) | 5 (8%) | 1 (2%) | 1 (2%) |
| 0 unchanged | 9 (14%) | 1 (2%) | 2 (4%) | 5 (9%) |
| +1 | 9 (14%) | 12 (19%) | 11 (20%) | 10 (17%) |
| +2 | 12 (19%) | 16 (25%) | 10 (18%) | 15 (26%) |
| +3 | 20 (32%) | 18 (28%) | 18 (33%) | 14 (24%) |
| +4 | 8 (13%) | 7 (11%) | 10 (18%) | 6 (10%) |
| +5 completely recovered | 0 | 0 | 0 | 3 (5%) |
| Valid n | 63 | 64 | 55 | 58 |
| *Missing (% of total)* | *7 (10%)* | *6 (9%)* | *15 (21%)* | *12 (17%)* |
| Total n | 70 | 70 | 70 | 70 |
| ^a^NSEIT; Neck-specific exercise with internet support  ^b^NSE; Neck-specific exercise at physiotherapy clinic | | | | |

**Adverse events**

Adverse events are presented in Table 4.

| **Table 4.** Number of participants reporting each negative effects, n (% of total) | | | | | | |
| --- | --- | --- | --- | --- | --- | --- |
|  | NSEIT ^a^ (n = 70) | | | NSE ^b^ (n = 70) | | |
|  | WAD II ^c^ | WAD III ^d^ | Total | WAD II ^c^ | WAD III ^d^ | Total |
| Neck pain | 7 | 5 | 14 (20%) | 3 | 8 | 11 (15%) |
| Dizziness | 4 | 1 | 5 (7%) | 2 | 2 | 4 (5%) |
| Headache | 4 | 1 | 7 (10%) | 3 | 0 | 6 (8%) |
| Arm pain | 0 | 2 | 2 (3%) | 3 | 0 | 3 (4%) |
| Nausea | 0 | 0 | 0 | 0 | 3 | 3 (4%) |
| Muscle soreness | 2 | 2 | 4 (5%) | 1 | 2 | 3 (4%) |
| Enhanced symptoms ^e^ | 3 | 1 | 4 (5%) | 7 | 5 | 12 (17%) |
| Musculoskeletal pain ^f^ | 1 | 1 | 3 (4%) | 2 | 0 | 2 (3%) |
| Low back pain | 1 | 1 | 2 (3%) | 0 | 0 | 0 |
| Total | 22 | 14 | 36 | 21 | 20 | 41 |
| ^a^NSEIT; Neck-specific exercise with internet support  ^b^NSE; Neck-specific exercise at a physiotherapy clinic  ^c^WAD II; Whiplash-associated disorders grade II  ^d^WAD III; Whiplash-associated disorders grade III  ^e^Exacerbation of symptoms when progression to exercise with rubber band (NSEIT) and gym exercises (NSE)  ^f^Pain > 2 weeks, symptoms decreased with slower exercise progression | | | | | | |
